# Unravelling electro-chemo-mechanical processes in graphite/silicon composites for designing nanoporous and microstructured battery electrodes

**DOI:** 10.1038/s41565-025-02027-7

**Published:** 2025-10-24

**Authors:** Xuekun Lu, Rhodri E. Owen, Wenjia Du, Zhenyu Zhang, Antonio Bertei, Roby Soni, Xun Zhang, Francesco Iacoviello, Daqing Li, Alice Llewellyn, Jianuo Chen, Han Zhang, Xuhui Yao, Qi Li, Yunlong Zhao, Shashidhara Marathe, Christoph Rau, Paul R. Shearing

**Affiliations:** 1 School of Engineering and Materials Science, Queen Mary University of London, London, UK; 2 The Faraday Institution, Quad One, Harwell Science and Innovation Campus, Didcot, UK; 3 Electrochemical Innovation Lab, Department of Chemical Engineering, UCL, London, UK; 4 The ZERO Institute, University of Oxford, Oxford, UK; 5 Department of Engineering, University of Exeter, Cornwall, UK; 6 Department of Civil and Industrial Engineering, University of Pisa, Pisa, Italy; 7 Henry Royce Institute, Department of Materials, University of Manchester, Manchester, UK; 8 WMG, University of Warwick, Coventry, UK; 9 National Physical Laboratory, Teddington, UK; 10 Beijing Key Laboratory of Heat Transfer and Energy Conversion, Beijing University of Technology, Beijing, China; 11 Dyson School of Design Engineering and Centre for Processable Electronics, Imperial College London, London, UK; 12 Diamond Light Source, Harwell Science and Innovation Campus, Didcot, UK

**Keywords:** Batteries, Materials for energy and catalysis, Materials science, Imaging techniques

## Abstract

Silicon is a promising negative electrode material for high-energy batteries, but its volume changes during cell cycling cause rapid degradation, limiting its loading to about 10 wt.% in conventional graphite/Si composite electrodes. Overcoming this threshold requires evidence-based design for the formulation of advanced electrodes. Here we combine multimodal operando imaging techniques, assisted by structural and electrochemical characterizations, to elucidate the multiscale electro-chemo-mechanical processes in graphite/Si composite negative electrodes. We demonstrate that the electrochemical cycling stability of Si particles strongly depends on the design of intraparticle nanoscale porous structures, and the encapsulation and loss of active Si particles result in excessive charging current being directed to the graphite particles, increasing the risk of lithium plating. We also show that heterogeneous strains are present between graphite and Si particles, in the carbon-binder domain and the electrode’s porous structures. Focusing on the volume expansion of the electrode during electrochemical cycling, we prove that the rate performance and Si utilization are heavily influenced by the expansion of the carbon-binder domain and the decrease in porosity. Based on this acquired knowledge, we propose a tailored double-layer graphite/Si composite electrode design that exhibits lower polarization and capacity decay compared with conventional graphite/Si electrode formulations.

## Abstract (web summary)

Multimodal operando imaging reveals how multiscale structural design affects lithiation heterogeneity and electrochemical cycling stability in graphite/silicon composite battery electrodes.

## Main

High-energy lithium-ion batteries (LiBs) are pivotal to the electric vehicle revolution. However, graphite, the state-of-the-art negative electrode active material in LiBs, has a limited theoretical capacity (372 mAh g⁻¹). Thus, battery scientists focused on silicon (Si), which offers nearly ten times higher theoretical capacity (3,579 mAh g⁻¹). However, the practical implementation of Si in LiB negative electrodes is hampered by rapid capacity fading associated with detrimental volume changes during charge–discharge cycling.

Nanostructured Si electrode active materials^1,2^ improve the cell’s specific discharge capacities, but their high surface area leads to excessive electrolyte consumption, continuous formation of the solid electrolyte interphase (SEI) and low Coulombic efficiencies of the cell. Moreover, their fabrication processes are cost-intensive. A more scalable strategy consists of blending micrometre-sized Si (μ-Si) with graphite, offering improved electrical conductivity, buffering of the Si volume expansion and electrode manufacturing compatibility. Yet, practical Si content is limited to ≤10 wt.% to guarantee adequate LiB’s cycle life^3,4^. Porous Si (refs. ^5,6^) is reported to mitigate volume expansion, but the fundamental understanding of nanoscale effects is limited. Three-dimensional (3D) heterogeneous graphite/μ-Si electrodes (that is, structurally and compositionally varied architectures) are also considered as a viable alternative to conventional design. However, the electro-chemo-mechanical processes of these 3D-architectured electrodes are not yet fully understood despite various recent studies^7–16^. Operando transmission electron microscopy captures atomic-scale electrochemical lithiation^17,18^ but lacks electrode-level insight. Macroscopic techniques (for example, dilatometry^19^ and fibre-optic sensing^20^) capture global expansion but miss local heterogeneity. Operando X-ray computed tomography (CT) combined with digital volume correlation (DVC)^21,22^ enables 3D visualization of morphological changes and local strain^11,23,24^, yet has rarely mapped these phenomena to the heterogeneous distribution of Si, graphite, carbon-binder domain (CBD) and electrode’s porosity. In particular, the role of CBD microstructure and phase-specific strain remains unclear and requires multimodal imaging for adequate investigations. Electrochemical techniques complement imaging^25,26^. For example, electrochemical impedance spectroscopy (EIS) measurements are essential for assessing interfacial and charge-transfer processes^27–30^, but their interpretation generally relies on an equivalent circuit modelling approach^31,32^, which can be ambiguous. For this reason, the distribution of relaxation times (DRT) method becomes popular for analysing raw EIS measurement data. Indeed, DRT assists in developing equivalent circuit models that are physically meaningful and less likely to be overparameterized^33–35^. In the case of Si-based electrodes, the DRT approach enables the distinction between SEI and charge transfer resistance and tracks their evolution during battery cycling^36^.

In this study, we present physics-driven, evidence-guided design of high-performance graphite/μ-Si composite electrodes. Operando optical microscopy coupled with digital image correlation (DIC) captures strain heterogeneity and competing lithiation dynamics at the particle and electrode levels. Operando synchrotron X-ray CT with DVC examines 3D microstructural evolutions, highlighting how the CBD and electrode porosity affect lithiation and strain. Five key materials design challenges are identified: (1) CBD trade-offs between capacity and electronic transport, (2) porosity trade-offs between capacity and ionic transport, (3) silicon loss leading to lithium plating, (4) graphite enclosure delaying silicon lithiation and (5) silicon expansion blocking electrolyte access. These insights inform the fabrication of a double-layer (DL) graphite/μ-Si composite electrode with more uniform lithiation, lower polarization and improved cycling performance (when tested against a lithium metal counter electrode using a Li-containing non-aqueous electrolyte solution) compared with conventional graphite/μ-Si composite electrode architectures.

## Investigating the lithiation and strain of graphite/Si electrodes via optical microscopy measurements

**Fig. 1:**
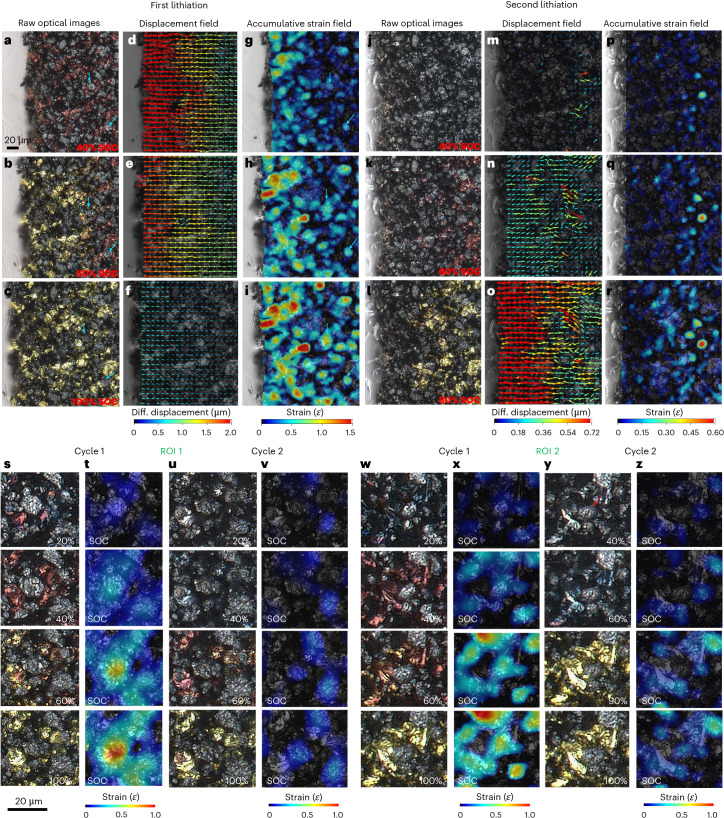
Operando optical microscopy of a graphite/μ-Si composite electrode (45 wt.% porous Si loading) during the first and second lithiation. **a**–**r**, The microstructural evolution at 40% SOC (**a**), 60% SOC (**b**) and 100% SOC (**c**) is shown alongside the differential (diff.) displacement field at 40% SOC (**d**), 60% SOC (**e**) and 100% SOC (**f**), and accumulative strain at 40% SOC (**g**), 60% SOC (**h**) and 100% SOC (**i**). **j**–**r**, The microstructural evolution at 40% SOC (**j**), 60% SOC (**k**) and 100% SOC (**l**) is shown alongside the differential (diff.) displacement field at 40% SOC (**m**), 60% SOC (**n**) and 80% SOC (**o**), and accumulative strain at 40% SOC (**p**), 60% SOC (**q**) and 80% SOC (**r**) as a function of charging for the second lithiation cycle (note the different scale bars of displacement and strain field between the first and second lithiation). Cyan arrows point at two representative Si particles experiencing volume expansion and fracture. **s**–**z**, In ROI 1, the evolution of particle morphology (**s**) and strain (**t**) during Cycle 1 is compared with the particle morphology (**u**) and strain (**v**) in Cycle 2. In ROI 2, the evolution of particle morphology (**w**), and strain (**x**) during Cycle 1 is compared with the particle morphology (**y**) and strain (**z**) in Cycle 2. The comparisons of the two ROIs reveal the competing reaction kinetics at the particle level as a function of SOC. Compared with ROI 1 (**s**–**v**), the central Si particle in ROI 2 (**w**–**z**) is tightly surrounded by the adjacent graphite particles.

**Extended Data Fig. 1:**
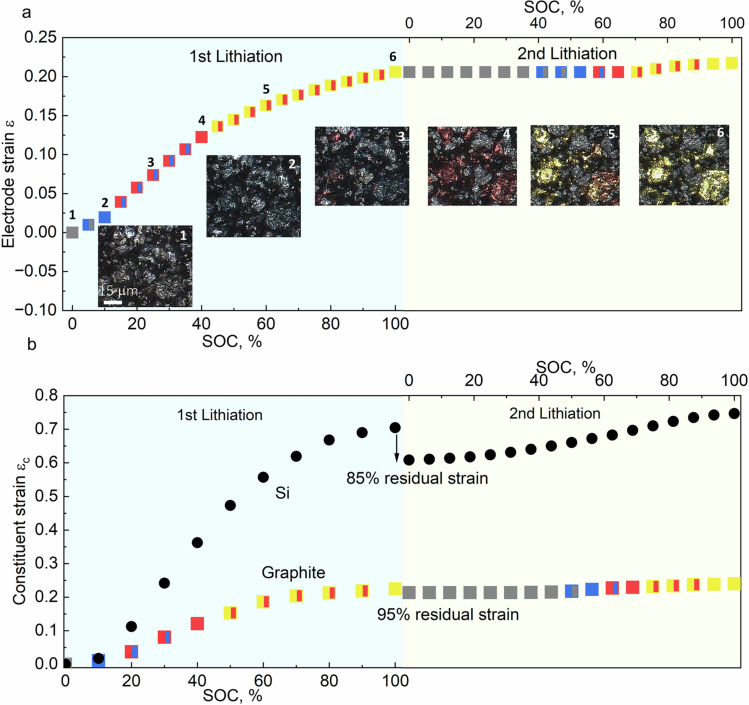
Strain evolutions at the electrode and particle scale. (**a**) Macroscopic electrode strain and (**b**) averaged strain in the Si and graphite particles as a function of the SOC in the 1^st^ and 2^nd^ cycles. The coloured symbols represent the stages of graphite particles.

The morphological evolution of a graphite/μ-Si composite working electrode (45 wt.% polycrystalline porous Si, 7–10 μm, particle porosity 0.35) is first visualized using operando optical microscopy (pixel size 0.15 μm) during lithiation at a current density of 2.5 mA cm^−2^, using Li metal as the counter electrode and a Li-containing non-aqueous electrolyte solution. The experimental set-up is detailed in Supplementary Note 1 and Supplementary Fig. 1. Full lithiation timesteps are shown in Supplementary Fig. 2. Strain maxima identified by DIC align closely with Si particles (Fig. 1a–c,g,h, cyan arrows). Substantial electrode expansion is observed and quantified in Extended Data Fig. 1a. Strain develops gradually until graphite reaches stage III (dark blue), then increases linearly as it transitions to stage II (red), slowing again at a high state of charge (SOC) owing to SOC gradients in the low-porosity/large-thickness electrode.

The differential displacement fields (Fig. 1d–f) show expansion aligned with the lithiation direction and a displacement gradient due to electrolyte transport limitations. This gradient can cause particle decohesion and disrupt electrical integrity. Expansion rate decreases with SOC (Fig. 1d–f). Accumulative strain maps (Fig. 1g–i) reveal maximum strain (up to 1.5) near the electrode edge, where SOC is highest. Strain is heterogeneous, with Si particles (cyan arrows) showing local maxima (0.75–1.5), far exceeding that in graphite (approximately 0.2). These phase-specific strains are decoupled and presented in Extended Data Fig. 1b (more details in Methods, Supplementary Figs. 3 and 4 and Supplementary Note 2). Both phases follow similar macroscopic trends, suggesting a coordinated and synchronous deformation during the first lithiation. High porosity (0.48) buffers large Si strain, limiting overall expansion of the composite electrode to 20%. At SOC >70%, reduced porosity and increased Si volume make Si’s contribution to global strain more pronounced, as indicated by the similar trend in the expansion curves of the electrode and Si (Extended Data Fig. 1a,b), where the expansion of graphite particles reaches a plateau. Full lithiation and strain development are shown in Supplementary Videos 1 and 2, respectively. Minimal lithium-ion exchange is seen during relaxation (Supplementary Note 3 and Supplementary Figs. 5 and 6), and some Si particles become electrochemically inactive in the second cycle (Supplementary Video 3) due to electrical disconnection, also reflected by low initial cell’s Coulombic efficiency (approximately 50%; Supplementary Fig. 7). This latter aspect is discussed in Supplementary Note 4.

Distinct lithiation kinetics are observed in the second lithiation (Fig. 1j–r and Supplementary Video 3): Si particles lithiate first, as shown by their heterogeneous expansion (Fig. 1j,m) preceding the colour change of the graphite particles. This shift is expected, because amorphous Si lithiates at higher potentials (0.4 V) than graphite (0.2 V)^3,37^. By 40% SOC, electrode expansion remains minimal due to reduced Si expansion—caused by residual strain (Extended Data Fig. 1b) and mechanical damage from the first cycle (Supplementary Fig. 9 and Supplementary Note 5). The mild localized Si strain is buffered by the static graphite matrix (that is, before lithiation onset) and accommodated by porosity recovery from the prior delithiation (Supplementary Figs. 8 and 9). The electrode tested in the optical cell shows limited global strain recovery, probably due to the absence of compressive constraints along the lithiation direction. After 50% SOC, strain develops in graphite (stage II, red), aligning with the onset of global expansion (Fig. 1k,n), implying its role as a mechanical backbone. These findings complement the results obtained from other techniques, such as dilatometry^19,38^ and X-ray diffraction^39–41^ that deconvolves the graphite and Si strains via Rietveld refinement. The strain evolution in the second cycle can be seen in Supplementary Video 4.

Local reaction kinetics between graphite and Si particles are examined in two regions of interest (ROIs) (Fig. 1s–z and Supplementary Fig. 10), where the central Si in ROI 2 is more tightly surrounded by graphite particles. After full delithiation, Si does not fully revert to its original size due to irreversible mechanical damage (Fig. 1u and Supplementary Note 5), leading to lower strain in the second cycle (Fig. 1v). Si expansion precedes graphite lithiation in the second cycle, but exceptions exist: in ROI 2, a central Si particle (Fig. 1y, red arrow) encapsulated by graphite remains inactive until 90% SOC (Fig. 1z and Supplementary Video 5), probably due to local electrolyte deficiency from densification. Li⁺ initially intercalates into graphite before accessing the Si particle, underscoring the influence of spatial arrangement on lithiation kinetics. This could cause Si underutilization and premature graphite overcharging. Notably, the graphite transition from stage III (SOC 0.23) to stage I (SOC 0.95) completes merely within a 30% SOC window (Fig. 1y), indicating current redistribution due to inactive Si—raising the local lithiation current in graphite and lithium plating risk.

**Fig. 2:**
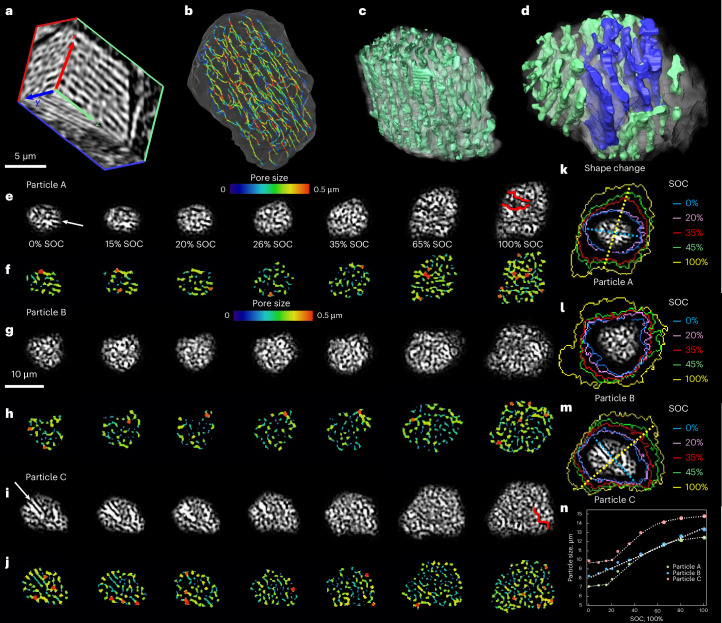
Microstructural evolution of three distinct porous Si particles. **a**, Visualization of the directional porosity within the single particle by X-ray nano-CT at the voxel resolution of 64 nm. **b**, Skeletonization of the internal porosity, with the colour map showing the pore size distribution. **c**,**d**, Three-dimensional volume rendering of two distinct types of pore structure, showing tubular porosity (**c**) and a combination of planar and tubular porosity (**d**). **e**–**j**, The microstructural evolution of particle A (**e**) is compared with particle B (**g**) and particle C (**i**). The corresponding pore size evolutions are compared between particle A (**f**), particle B (**h**) and particle C (**j**). Both particle A (**e**) and particle C (**i**) contain heterogeneous linear porous structures but with different orientations, whereas particle B (**g**) has homogeneous porosity. The evolution of particle contours and the long axis orientations are compared between particle A (**k**), particle B (**l**) and particle C (**m**). **n**, The change of particle size as a function of the SOC for the three particles. Red lines in **e** and **i** at 100% SOC highlight the crack formation.

X-ray nano-CT (voxel size 64 nm) reveals that the commercial µ-Si particles (supplied by E-magy) possess unidirectional tubular porosity (Fig. 2a) formed during the material synthesis through a directional solidification process. Pore centrelines and size distribution are visualized in Fig. 2b, with 3D porosity rendering in Fig. 2c. Some particles also show mixed morphologies—tubular (green) and planar (blue) porosity—as seen in Fig. 2d and confirmed by scanning electron microscopy images (Supplementary Fig. 11). To assess how nanoporous morphologies affect cycling stability, three Si particles were tracked via operando optical imaging. Particle A, with horizontal tubular pores (Fig. 2e, *xz* plane), transitions from linear to uniform punctate porosity by 26% SOC. This reorganization, with decreased pore size and porosity (Fig. 2f), accommodates volume change and indicates a crystalline-to-amorphous transition. After 26% SOC, the transformation from heterogeneous to homogeneous porosity is complete. Further lithiation causes marked expansion and new pore formation. A major crack is observed at 100% SOC (highlighted in red). Particle B, with punctate pores (Fig. 2g, *xy* plane), shows stable morphology and no cracking. Particle C combines punctate and linear porosity (Fig. 2i, white arrow), with its 3D morphology shown in Fig. 2d. Linear regions undergo similar reorganization and cracking as particle A. The thickness maps demonstrate that the pore size and porosity increase in all three particles at high SOCs.

Moreover, particles A and C exhibit highly anisotropic expansion, with maximum swelling perpendicular to their tubular pore orientation, while particle B (with punctate porosity) expands isotropically in the *xy* plane. This difference is visualized in Fig. 2k–m, with overlapping contours (blue and pink) indicating minimal expansion below 20% SOC. Figure 2n quantifies a three-stage expansion in particles A and C: (i) pore reorganization (0–20%), (ii) linear expansion (20–60%) and (iii) reduced expansion beyond 60% SOC, probably due to cracking-induced transport limitations. Particle B expands nearly linearly. These trends highlight how pore geometry influences the electrochemical cycling stability of Si particles: tubular porosity enables controlled swelling, while planar porosity leads to uneven, anisotropic expansion and cracking. A capped SOC of 60–70%, corresponding to the inflection point after linear expansion in Fig. 2n, is recommended to preserve structural integrity and prolong the cycle life.

## Microstructural evolution and spatial dynamics in the graphite/Si composite electrodes via X-ray CT

While operando optical microscopy offers high-resolution insight into intra- and interparticle processes, synchrotron X-ray CT complements it with 3D visualization and clear phase contrast (voxel size 0.325 μm; Supplementary Fig. 12). Here, three different graphite/Si composite electrodes (two graphite/μ-Si composite electrodes with different Si loadings and one graphite/μ-SiO_*x*_ composite electrode) are compared to elucidate the interplay between microstructure, physical and electrochemical performance.

**Fig. 3:**
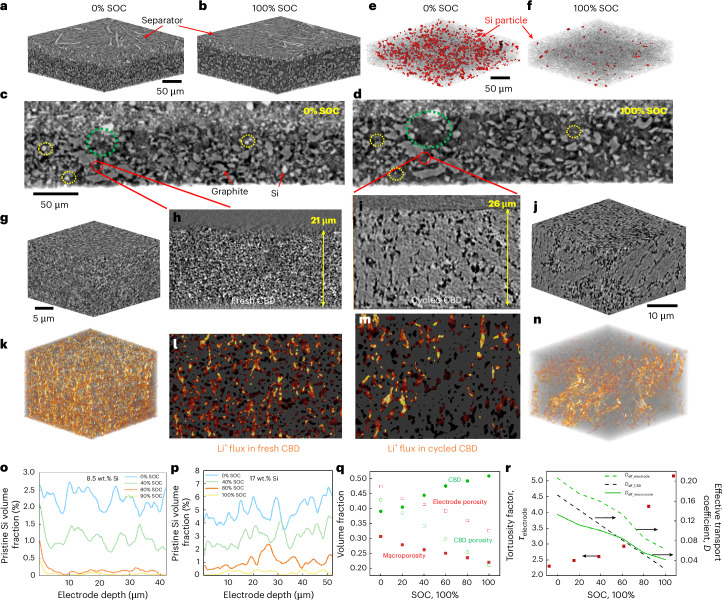
Three-dimensional microstructural evolution of the graphite/μ-Si composite electrodes (8.5 and 17 wt.% Si loading) at 0.4 mA cm^−2^ lithiation current by synchrotron X-ray CT. **a**–**d**, The volume expansion is compared between uncharged (**a**) and fully charged (**b**) states. One representative slice is compared between the uncharged (**c**) and fully charged (**d**) states. The dotted yellow circles in **c** and **d** show the drastic greyscale change of the Si particles after lithiation; the dotted green circles highlight the drastic local expansion arising from the CBD. **e**,**f**, Visualization of the spatial distribution of the pristine and unlithiated Si particles before (**e**) and after (**f**) charge. **g**–**j**, Comparison of the morphological change of the CBD in 3D before (**g**) and after (**j**) cycling and in a 2D cross-sectional slice before (**h**) and after (**i**) cycling in Li||CBD laboratory-scale cell. **k**–**n**, Visualization of the Li^+^ ion flux distribution in the 3D at fresh (**k**) and cycled (**n**) state and a 2D cross-sectional slice of the reconstructed porous CBD at fresh (**l**) and cycled (**m**) state. **o**,**p**, Comparison of the utilization of Si particles by reporting the volume fraction of pristine Si along the depth direction as a function of SOC for the composite electrode with 8.5 wt.% (**o**) and 17 wt.% (**p**) Si loading. **q**, Plot of the variation of macroporosity, CBD porosity, electrode porosity and volume fraction of CBD as a function of the SOC. **r**, Quantification of the evolution of the tortuosity factor of the electrode, alongside the effective transport coefficient for macropores, CBD and the electrode (see details in the Methods). Source data

Figure 3a,b show the 3D microstructure of a graphite/μ-Si electrode (5.7 mAh cm^−2^, 17 wt.% Si) before and after charging, revealing approximately 20% thickness expansion and increased interparticle spacing (Supplementary Video 6). Cross-sections (Fig. 3c,d) illustrate crystalline Si (white) transforming into amorphous (dark grey), becoming indistinguishable from CBD (yellow circles). Most Si particles lithiate (Fig. 3e,f), though some remain pristine. Notably, CBD expansion (20 wt.%) remarkably contributes to electrode swelling (Fig. 3c,d, green circles). This feature is not caused by any unresolved submicrometre Si particles within the CBD (Supplementary Note 6). To support this, X-ray nano-CT (voxel resolution 0.064 μm) of a Li||CBD laboratory-scale cell (Fig. 3g–j; potential profile in Supplementary Fig. 13) displays approximately 25% CBD thickening, accompanied by coarsening and porosity loss—linked to SEI formation and electrolyte uptake^42^. This causes reduced Li⁺ flux and connectivity (Fig. 3k–n). However, CBD expansion is not observed upon electrolyte solution immersion alone (Supplementary Fig. 14). Figure 3o,p compares Si utilization between the 8.5 wt.% and 17 wt.% Si composite electrodes, whereas Fig. 3q,r summarizes the microstructural evolution of the latter, which are discussed in Supplementary Note 7.

**Fig. 4:**
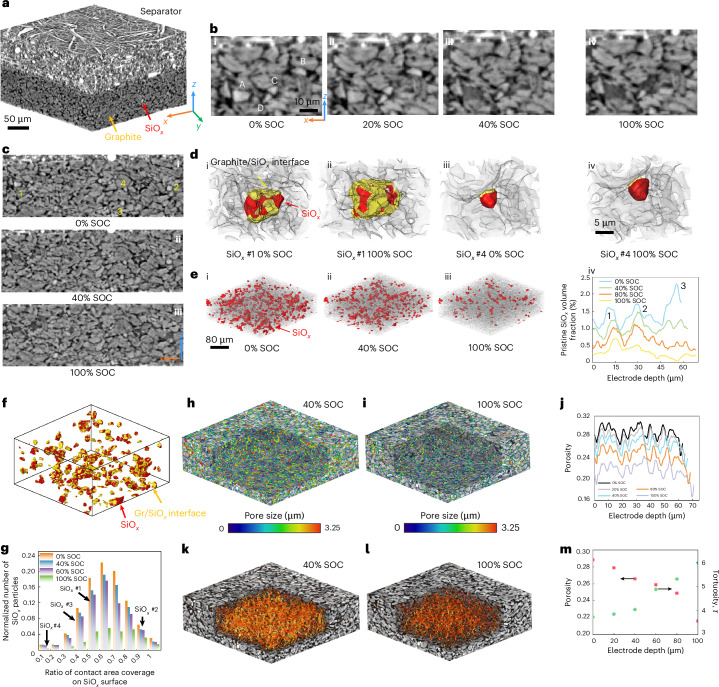
Three-dimensional microstructural evolution and heterogeneous lithiation in a densely packed graphite/μ-SiO_*x*_ composite electrode during cycling at 0.3 mA cm^−2^. **a**, A 3D volume rendering of the electrode and separator. **b**, A ROI analysis of the local lithiation heterogeneity between four representative SiO_*x*_ particles. **c**, Lithiation heterogeneity along the thickness direction as a function of SOC. **d**, A 3D rendering of the local microstructural environment of two SiO_*x*_ particles with the fastest and slowest lithiation reaction activity, respectively (yellow: contact interface between graphite and SiO_*x*_). **e**, The spatial distribution of the pristine SiO_*x*_ particles ((i)–(iii)) and the variation of its volume fraction as a function of the SOC ((iv)). **f**, The spatial distribution of the pristine SiO_*x*_ particles (red) and the graphite–SiO_*x*_ contact interface (yellow). **g**, The distribution of SiO_*x*_ particles with varying ratio of the contact surface area to the total area of the particle at increasing SOC. Each column is normalized relative to the total number of Si particles in the pristine state. **h**,**i**, The spatial distribution of the porosity at 40% (**h**) and 100% (**i**) SOC. **j**, Quantified variation of the porosity along the thickness direction (*x* = 0 at the electrode–separator interface). **k**,**l**, Visualization of the Li^+^ ion flux simulated within the 3D reconstructed porosity at 40% (**k**) and 100% (**l**) SOC. **m**, The variation of electrode porosity and tortuosity factor as a function of SOC. Source data

However, lithiation dynamics differ markedly in a densely packed graphite/μ-SiO_*x*_ composite electrode (4.5 mAh cm^−2^, 5 wt.% SiO_*x*_) collected from a commercial 3.35 Ah INR18650-MJ1 cell, which has lower porosity (0.29) and trace CBD content. The 3D reconstructed volume is shown in Fig. 4a. An ROI near the separator containing four SiO_*x*_ particles (A, B, C and D) is shown in Fig. 4b(i)–(iv). Surprisingly, particle A (∼10 μm) lithiates first (indicated by the change of contrast in Fig. 4b(iii)) despite being larger than C and D. Particle B, with less graphite contact and closer to the separator, lithiates after A, confirming that electrical connectivity dominates over electrolyte access. At 100% SOC, particle D remains inactive (Fig. 4b(iv)), displaying strong lithiation heterogeneity. Figure 4c(i)–(iii) shows this heterogeneity across the *x*–*z* plane as SOC increases. Along depth, only the largest particle 1 reacts at 40% SOC (Fig. 4c(ii)); by 100% SOC, particles 2 and 3 are lithiated, while particle 4 remains inactive. The delayed lithiation of particle 2 is probably due to graphite enclosure hindering electrolyte access—consistent with ROI 2 in Fig. 1y,z. The full dynamic evolution is shown in Supplementary Video 7.

The local microstructures are analysed to reveal the cause of the reaction heterogeneity between particle 1 (fastest) and particle 4 (slowest) (Fig. 4d(i)–(iv)). Particle 1 has an extensive SiO_*x*_–graphite contact area (yellow voxels), which augments during lithiation, fostering electronic transport despite its distance from the current collector. Particle 4 remains physically isolated (Fig. 4c(i)–(iii)), limiting its lithiation kinetics. Thus, both initial contact area and its evolution—alongside particle enclosure—drive heterogeneity. Pristine SiO_*x*_ versus SOC is visualized in Fig. 4e(i)–(iii). Near the separator, more underutilized SiO_*x*_ particles are limited by electron transport (Fig. 4e(iv) and Supplementary Note 8). The SiO_*x*_ particles (red) and the graphite–SiO_*x*_ contact areas (yellow) are visualized in Fig. 4f and summarized in Fig. 4g. Most particles exhibit 0.4–0.8 surface coverage. Particles outside this range show minimal lithiation up to 60% SOC: low-contact particles lack electrons; high-contact ones are ion-blocked by graphite encapsulation. At 100% SOC, many top-half SiO_*x*_ particles remain electrochemically inactive despite the good surface contact ratio between 0.4 and 0.8, caused by the volume expansion-induced disruption of electron-transport pathways formed by graphite particles (65 vol.% backbone), as the interfacial contact between graphite particles is reduced by 50% at 100% SOC (Supplementary Fig. 15). Figure 4g,h visualizes the size distribution of the porosity at 40% and 100% SOC, with quantitative analysis provided in Fig. 4i. This leads to deteriorating electrolyte transport and is characterized in terms of the resultant Li^+^ ion flux in the pore phase (Fig. 4j,k), and the volume-averaged porosity/tortuosity factor (Fig. 4l) as a function of SOC.

## Microstructure-driven multiscale strain evolutions in graphite/Si composite electrodes

**Fig. 5:**
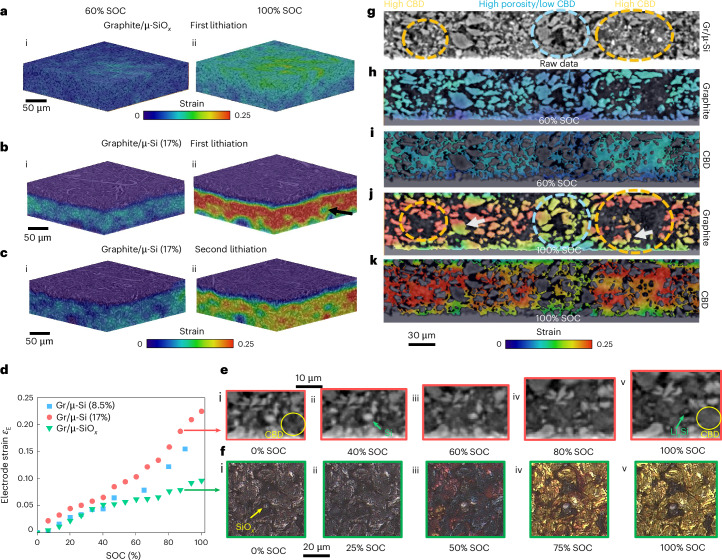
Correlating the volumetric strains at the global and local scale with microstructural heterogeneities. **a**–**c**, Visualization of the accumulative strain in the graphite/μ-SiO_*x*_ electrode, as well as the first and second lithiation of the graphite/μ-Si electrode (17 wt.% Si). **d**, Comparison of the distinct expansion rate of the three types of electrode with SOC during the first lithiation process. **e**, Local ROI analysis reveals the morphological change and expansion of CBD. **f**, The expansion of SiO_*x*_ particles saturated within 50% SOC observed by operando optical microscopy. **g**, The cross-section of the graphite/μ-Si (17 wt.%) electrode by X-ray CT, which shows the variations of the locally high CBD and porosity regions. **h**,**i**, The volumetric strain in graphite (**h**) and CBD (**i**) phase at 60% SOC. **j**,**k**, The volumetric strain in graphite (**j**) and CBD (**k**) phase at 100% SOC. Note that the separator in the graphite/μ-SiO_*x*_ electrode in **a** and **b** is hidden to better visualize the strain gradient from the electrode–separator interface into the deeper regions. Source data

**Extended Data Fig. 2:**
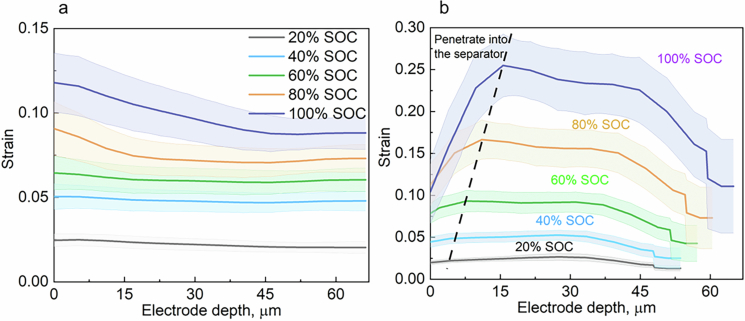
In-plane electrode strain across the thickness direction as a function of SOC. (**a**) graphite/μ-SiO_x_ electrode; (**b**) graphite/μ-Si electrode (1^st^ lithiation). The shaded areas represent the magnitude of in-plane variation.

Figure 5a–c compares the volumetric strain measured by DVC technique^43^ in the graphite/μ-SiO_*x*_ and graphite/μ-Si (17 wt.% Si) composite electrodes. The graphite/μ-SiO_*x*_ electrode exhibits relatively homogeneous strain, except near the separator, where higher strain correlates with electrolyte concentration gradients in the dense structure (porosity 0.29; Fig. 5a(i)–(ii)). This gradient is mild at 0.3 mA cm^−2^ but becomes pronounced at 4.5 mA cm^−2^ (Supplementary Fig. 16). By contrast, the graphite/μ-Si electrode exhibit highly heterogeneous and larger strain across graphite, Si, and CBD (Fig. 5b,c). Some particles near the current collector remain electrochemically inactive due to delamination (Fig. 5b(ii); see evidence in Supplementary Fig. 17). A porous graphite particle exhibits a relatively low strain (Fig. 5b(ii), black arrow), suggesting internal porosity buffers expansion. In the second cycle, strain at 100% SOC decreases (Fig. 5c(ii)), primarily due to structural damage (that is, cracking), degradation and loss of active Si particles, which result in high residual strain and reduced capacity following the first cycle, as observed in the optical microscopy measurements. The porous graphite particles also exhibit lower strain in the second cycle (Extended Data Fig. 1b and Supplementary Fig. 9). Distinct strain versus depth profiles are shown in Extended Data Fig. 2 and discussed in Supplementary Note 9.

All three electrodes exhibit a two-stage expansion profile macroscopically (Fig. 5d). In graphite/μ-Si electrodes, expansion rate increases beyond approx. 50% SOC. The underlying mechanism is elucidated through ROI analysis of the X-ray CT data (Fig. 5e). The macroporosity (black voxels) and the nanoporosity within the CBD (dark grey) are progressively filled by the expansion of CBD (yellow circle) and Si (green arrow) by 40% SOC, explaining the steady, linear global expansion below 50% SOC, primarily driven by the volumetric dominant CBD and graphite (65 vol.%). Si (approximately 5 vol.%) plays a local role early on, evident from the amorphous Li_*x*_Si shell around shrinking crystalline Si (Extended Data Fig. 4). Hence, varying Si loading (8.5 versus 17 wt.%) has little effect on early-stage expansion, consistent with the graphite/μ-SiO_*x*_ electrode’s strain despite different particle-level behaviour (70% versus 22% strain for Si versus SiO_*x*_). Above 50% SOC, expansion rises sharply as porosity is exhausted (Supplementary Video 8). Notably, the commercial graphite/μ-SiO_*x*_ electrode shows a reduced strain rate beyond 50% SOC, probably due to increased lithiation gradient (Extended Data Fig. 2a) and the early completion of SiO_*x*_ particle expansion, as shown in Fig. 5f and Supplementary Fig. 18, although a few particles with poor reactant access continue lithiation beyond this point.

**Extended Data Fig. 3:**
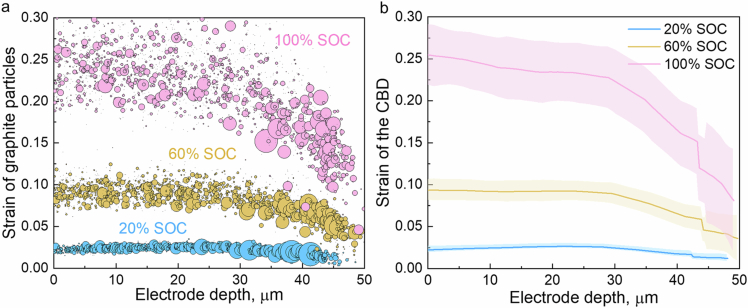
Volumetric strain along the thickness direction measured by DVC. (**a**) Strain of the individual graphite particles as a function of SOC, with symbol size proportionally representing the particle size; (**b**) strain in the CBD as a function of SOC, with shaded area indicating the in-plane variation.

**Extended Data Fig. 4:**
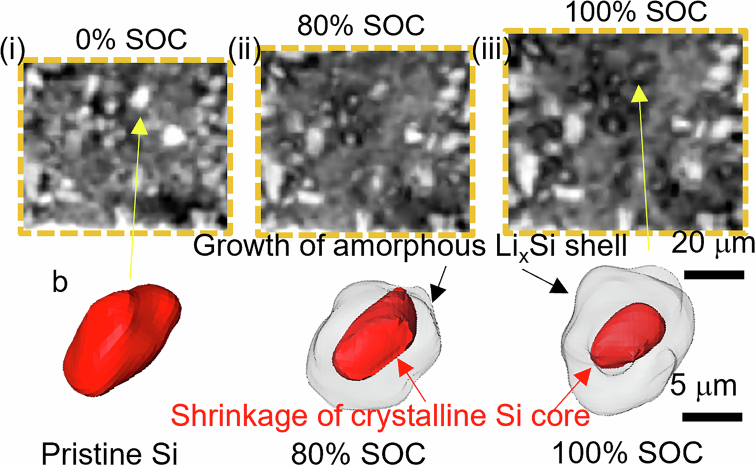
Morphological evolution of Si particles during lithiation. (**a**) X-ray CT data showing the microstructural evolution as a function of SOC. (**b**) 3D morphological evolution of the single Si particle. The remaining crystalline Si core (red) indicates capacity underutilization.

The impact of heterogeneous porosity and CBD on the reaction and volumetric strain is visualized in Fig. 5g–k. CBD-rich, low-porosity regions (dashed orange circles) exhibit higher graphite strain than CBD-poor, high-porosity areas (blue circles) at 60% SOC (Fig. 5h,i), and becomes more serious at 100% SOC (Fig. 5j,k). This suggests that the CBD that ensures percolated electronic contact plays a critical role in lithiation homogeneity. Near the current collector, strain is lower due to limited electrolyte access, exacerbated by reduced nano- and microporosity from CBD and active material expansion. Notably, intraparticle strain separation (Fig. 5j, white arrows) is observed in less lithiated graphite, arising from its phase separation phenomenon^44^. In-plane and through-thickness strain variations are detailed in Extended Data Fig. 3 and Supplementary Note 10. However, Si strain could not be reliably measured due to greyscale changes during lithiation (Extended Data Fig. 4a), which obscure boundaries and compromise DVC algorithm^21^. This aspect may affect CBD segmentation (Supplementary Note 11 and Supplementary Fig. 19). Remaining crystalline Si cores (Extended Data Fig. 4b) indicate incomplete lithiation, probably due to limited reactant access in dense CBD regions.

## Evidence-informed microstructural and compositional design of graphite/Si composite electrodes

**Fig. 6:**
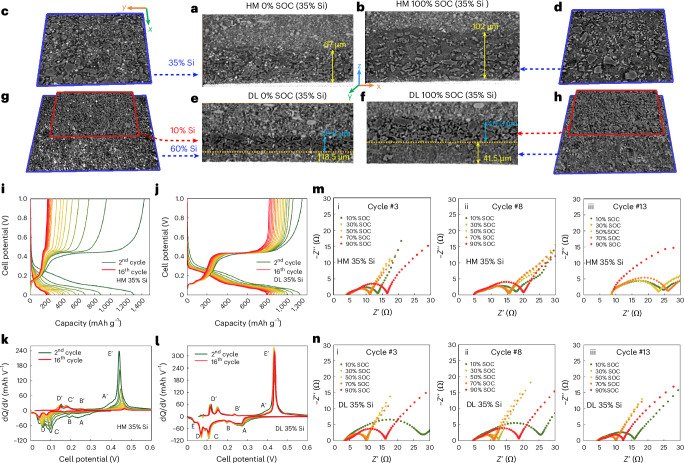
Comparison of the physical and electrochemical performance between the DL electrode and a reference homogeneous (HM) graphite/μ-Si composite electrode tested in Li metal coin cells. **a**–**d**, The microstructural evolution of the HM electrode in the same *x*–*z* plane at 0% SOC (**a**) and 100% SOC (**b**) and *x*–*y* plane at 0% SOC (**c**) and 100% SOC (**d**). **e**–**h**, The microstructural evolution of the HM electrode in the same *x*–*z* plane at 0% SOC (**e**) and 100% SOC (**f**) and *x*–*y* plane at 0% SOC (**g**) and 100% SOC (**h**) at a 0.45 mA cm^−2^ lithiating current. Note that the slices in red and blue frame in **g** and **f** represent the microstructure in the graphite-rich top layer and Si-rich bottom layer, respectively. **i**,**j**, The cyclic performance of the Li||HM (**i**) and Li||DL (**j**) coin cells galvanostatically cycled at 0.9 mA cm^−2^ and 25.8 °C (for sake of clarity, the first formation cycle, in which a large flat plateau corresponding to the crystalline to amorphous transition of Si particles, is excluded). **k**,**l**, The differential capacity plotted against the cell potential (dQ/dV versus V) for Li||HM (**k**) and Li||DL (**l**) coin cells. **m**,**n**, EIS spectra at the 3rd, 8th and 13th lithiation cycle for the Li||HM (**m**) and Li||DL (**n**) coin cells. Source data

The key challenges for material design in graphite/μ-Si composite electrodes based on our findings are the following: (1) low CBD boosts energy content of the cell but limits Si utilization, while high CBD causes severe expansion and porosity loss, degrading rate performance; (2) high porosity fosters electrolyte transport but sacrifices energy and electrical contact/percolation; (3) Si loss during cycling overloads nearby graphite, which is risky and may trigger early lithium plating; (4) tight graphite enclosures delay Si lithiation, lowering its activity; and (5) Si expansion obstructs electrolyte access to deeper regions. Building on these insights, we develop a heterogeneous DL graphite/μ-Si composite electrode, in which the top layer (separator side) consists of 82 wt.% graphite + 10 wt.% Si + 8% CBD (3% binder + 5% C45) and the bottom layer consists of 20 wt.% graphite + 60 wt.% Si + 20 wt.% CBD (10% binder + 10% C45). The total Si content is 35 wt.%. A homogeneous reference electrode (HM) with identical composition is also prepared (51 wt.% graphite + 35 wt.% Si + 14 wt.% CBD (7% binder + 7% C45)). Figure 6a–d and Fig. 6e–h present a comparison of the morphological evolution of the same cross-sectional slice in the HM and DL electrode, respectively, obtained by operando X-ray CT, before and after charging at 0.45 mA cm^−2^ in a non-aqueous Li metal coin cell configuration.

The DL electrode exhibits a lower initial thickness due to the thinner, Si/CBD-rich bottom layer, which undergoes two cycles of drying and shrinkage processes and is more prone to shrinkage due to the smaller particle size. Most Si particles undergo lithiation, evident from the greyscale shift from white to grey (Fig. 6a,c versus Fig. 6b,d, and Fig. 6e,g versus Fig. 6f,h). However, some grainy Si remains underlithiated (Fig. 6d,h), as shown in Supplementary Fig. 20. Supplementary Fig. 20e,f reveals that Si capacity in the DL electrode mainly originates from the bottom layer, while in HM, lithiation is more evenly distributed, although a mild gradient is visible due to limited porosity, which may become more serious in the following cycles. Thickness expansion in HM is less (52%, 67–102 μm) than that in DL (73%, 47.3–81.8 μm) because the latter preserves top-layer porosity (Fig. 6f) to ensure electrolyte access to the bottom layer; by contrast, the porosity in HM is largely filled (Fig. 6b).

**Extended Data Fig. 5:**
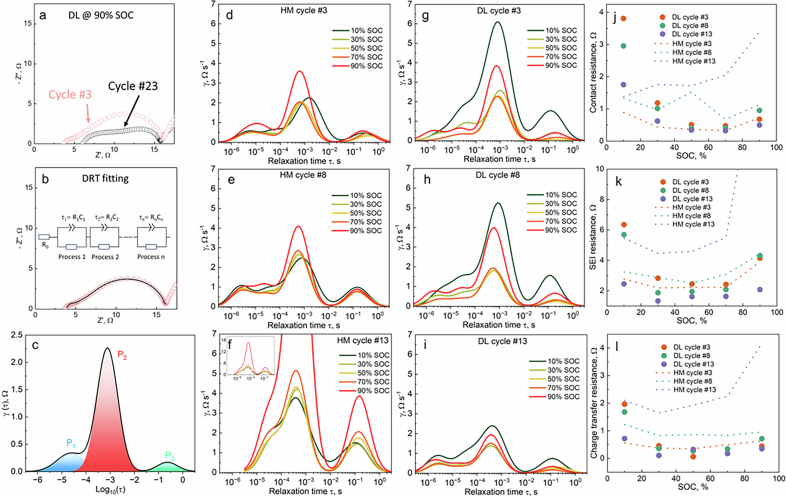
Distribution of Relaxation Times (DRT) analysis comparing the evolution of polarization processes for the Li | |HM and Li | |DL coin cells. The EIS spectrum of the 3^rd^ cycle and 23^rd^ cycle is shown in (**a**), and the equivalent circuit fitting of the raw EIS data with an infinite series of RC elements is shown in (**b**); (**c**) three distinct polarization peaks are obtained by DRT fitting of the EIS raw data; (**d**)-(**f**) and (**g**)-(**i**) compare the evolution of DRT peaks as a function of SOC and cycle number for the Li | |HM and Li | |DL coin cells, respectively; the resultant resistance for different polarization processes in the two different Li metal coin cells, by integrating the area under each peak, are quantified in (**j**)-(**l**).

Electrochemical cycling data of Li metal cells at 0.9 mA cm^−2^ comprising DL and HM working electrodes are reported in Fig. 6i,j (for visual clarity, the first formation cycle, marked by a large crystalline-amorphous transition plateau, is not reported in Fig. 6i,j). A faster capacity fade is observed in the cell with the HM electrode at the end of 16th cycle (72% versus 15%; Fig. 6j). The Li||HM cell shows severe capacity fade from both graphite and Si, evident from the reduced area of all cathodic (lithiation) peaks in differential capacity curves (Fig. 6k), especially A′ and E′, indicating Si loss (see interpretations in Supplementary Note 12). Peak shifts (B, C and D to lower potentials; E′ to higher) also suggest increased polarization. These effects are minimal in the Li||DL cell (Fig. 6l), indicating a reduced microstructural and electrochemical degradation. Analysis of the EIS measurements (Fig. 6m,n) further substantiate this. Indeed, in the Li||HM cell, total impedance (*R*_ohm _+ *R*_sei _+ *R*_ct_) rises from 11 to 23 Ω by the 13th cycle (50% SOC), while the one of the Li||DL cell remains stable (approximately 10 Ω), increasing slightly to 14 Ω by the 23rd cycle (Supplementary Fig. 21). DRT analysis reveals stable contact resistance and even a reduction in charge transfer and SEI resistance (Extended Data Fig. 5), attributed to improved local electrical contact, enhanced electrolyte accessibility in the bottom layer, better structural integrity provided by the graphite-rich top buffer layer and an increased parallel-connected reaction surface. Detailed interpretation is provided in Supplementary Note 13. This highlights the effectiveness of the DL composite electrode design to suppress the mechanical, microstructural and electrochemical degradation during battery operation at the Li metal coin cell level.

## Conclusions

This study provides new insights into 3D microstructural and strain evolutions, spatial dynamics of heterogeneous lithiation and performance degradation in graphite/μ-Si composite electrodes, assisted by multimodal multiscale operando imaging and advanced characterization techniques.

Anisotropic expansion and early cracking occur in Si particles with planar, heterogeneous nanoporosity, whereas those with homogeneous, tubular nanoporosity exhibit isotropic strain and structural integrity. Simultaneous Si and graphite deformation is observed during the first lithiation, whereas it is asynchronous in the following cycles. The impact of Si particle expansion on global strain is less pronounced at low SOCs, buffered by internal nanoporosity and static graphite particles (from the second cycle), but accelerates at high SOCs (>50%) due to electrode porosity exhaustion—driven largely by 20% CBD expansion. Structural damage causes a high residual strain of Si particles (75–85%), leading to trapped lithium and loss of active material. A capped SOC (up to 80%) is necessary to limit particle damage. Microstructural heterogeneity influences both reaction kinetics and mechanics. Graphite-encapsulated Si lithiates slowly or remains inactive. High CBD/low electrode porosity correlates with higher lithiation/strain. Although counter-intuitive, larger Si particles can lithiate faster than smaller ones provided adequate electronic contacts. A Si–graphite surface contact ratio of 0.4–0.8 is suggested as optimal to effectively balance electron and ion transport for efficient lithiation.

Our findings identify five major challenges in the microstructural design: (1) low CBD boosts energy content of the composite electrode in Li metal cell configuration but limits Si utilization, while high CBD causes severe expansion and porosity loss; (2) high electrode porosity fosters electrolyte transport but sacrifices energy and electrical percolation; (3) Si loss during cycling overloads nearby graphite, which is risky and may trigger early lithium plating; (4) tight graphite enclosures delay Si lithiation, lowering its activity; and (5) Si expansion obstructs electrolyte access to deeper regions of the electrode.

To overcome these issues, we design a DL heterogeneous graphite/μ-Si composite electrode (35 wt.% Si), featuring a porous, graphite-rich, CBD-poor top layer (towards the separator) and a Si-rich, CBD-rich bottom layer. This architecture provides four key advantages: (1) spatial separation mitigates graphite overcharging and releases encapsulated Si; (2) the porous top layer maintains fast electrolyte transport; (3) the graphite-rich top buffers expansion from the Si-rich bottom, while compressive stress preserves the structural and interfacial stability of Si particles; and (4) the high CBD content in the bottom layer ensures robust electrical contact during cycling. Indeed, this design improves capacity retention compared with conventional electrodes tested in a laboratory-scale non-aqueous Li metal cell configuration (72% versus 15% over 16 cycles).

In summary, this study reveals the impact of intraparticle nanoporosity, CBD evolution, short- and long-range 3D architectures on the lithiation heterogeneity and cyclic stability of the graphite/μ-Si composite electrode. Despite the low technology readiness level^45^, the proof of concept of the DL composite electrode design tested in Li metal coin cell configuration proposed in this work has shown promising results. Further optimization towards high-potential Li-ion cell architecture and evaluation of material scalability are required before this electrode design can be considered a viable candidate for practical large-area Li-ion batteries^46^.

## Methods

### Materials and electrode preparation

For the homogeneous composite electrode, 2 wt.% carboxymethyl cellulose binder (CMC, molecular weight *M*_w_ = 90,000, degree of substitution DS: 0.7, Sigma-Aldrich) was first added into a buffer solution with pH 3 consisting of deionized water (Sigma-Aldrich, conductivity ≤4.3 μS cm^−1^ at 25 °C), citric acid (Sigma-Aldrich, purity ≥99.5%) and KOH (Sigma-Aldrich, 45 wt.% in H_2_O). After sonicating (CREWORKS) and stirring (ANZESER Magnetic Stirrer) at 25.8 °C in air for at least 60 min, 10 wt.% conductive carbon (C45, Timcal) was added into the slurry and sealed in a jar for 30-min mixing at 25.8 °C in air using a planetary centrifugal mixer (THINKY ARE-250); subsequently, graphite powder (LiFun Technology, average particle size 17.09 μm, purity 99.99%) was added into the slurry and mixed at 2,000 rpm for 15 min, followed by the addition of μ-Si powder (particle size 7–10 μm, purity >99%, supplied by E-magy for optical measurements, and SILGRAIN Elkem ((D50: 5 µm; 50% of the particle is smaller than 5 µm), purity >98.25%) for X-ray CT), which was also mixed at 2,000 rpm for 15 min. The second binder styrene-butadiene rubber (SBR, Targray) was then added into the slurry (CMC/SBR ratio 2:1) and mixed at 2,000 rpm in Thinky mixture for 15 min. On the automatic coating machine, the slurry was then coated onto a 15-μm copper foil (MTI, purity ≥99.8%, non-dendritic, cleaned by isopropanol wipes after being attached to the coating plate) using a doctor blade with a gap distance of 250 μm to obtain a final electrode thickness of 90 μm (±2 μm), after drying at 60 °C using the built-in heating function on the coating bench for 30 min in air atmosphere, which was then calendered to the desired thickness using lab roller calendaring machine (MTI). Specifically, the electrode coating was passed through the gap between a rotating roller and a plate, which was collected at the outlet and fed through the gap again to ensure a uniform thickness. A gradual calendering process with three incremental steps was used: an initial large gap corresponding to 30% of the targeted thickness reduction, followed by a medium gap at 60% and concluding with a final gap achieving 100% of the reduction. At each intermediate and final step, the thickness was measured by a benchtop thickness gauge (DML).

For the DL electrode, the Si-rich bottom layer was coated using the same procedure without calendering; subsequently, the graphite-rich top layer was coated onto the dried bottom layer. The two layers were calendered together after drying. The mass loadings of Si for different electrodes used in this study have been specified in the corresponding context. Pure-CBD electrode coatings were prepared following the same procedure as for the graphite/Si composite electrodes, except that 90 wt.% C45 carbon was dispersed in a 10 wt.% binder solution (CMC + SBR) without graphite or Si. Owing to the fine particle size of C45, the slurry was coated using a 100-µm doctor blade gap, and no calendering was applied.

The graphite/SiO*ₓ* composite electrode was harvested from a commercial 3.35 Ah INR18650-MJ1 cylindrical cell. The cell was first discharged to 2.0 V using a constant current–constant potential (CC–CP) protocol at C/20. In an Ar-filled glovebox (H_2_O < 0.5 ppm, O_2_ < 0.5 ppm), the metallic casing was carefully cut open and peeled off from the positive terminal with an insulated cutter, and then the spirally wound electrodes and separator were unrolled. The positive electrode, negative electrode and separator were separated and rinsed twice in dimethyl carbonate (DMC, 2 min each) to remove residual electrolyte, followed by overnight drying inside the glovebox. On the negative electrode, the unwanted coating from one side of the double-sided Cu foil was removed by gently wiping with lint-free tissue moistened with isopropanol and deionized water. Finally, 15 mm discs of the single-sided graphite/SiO*ₓ* composite electrode were punched out using a precision disc cutter.

### Operando optical microscopy

A rectangular stripe of working electrode (9 mAh cm^−2^, the mass ratio of Si, graphite, C45 and binder is 45:45:5:5, 2 × 10 mm^2^), Li metal counter electrode (MTI, 99.9% lithium, 250 μm, diameter 15.6 mm), 260-μm-thick glass fibre separator (Whatman, 10 mm diameter, 1.6 µm pore size) and other components were assembled into an ECC-Opto-Std optical cell (EL-cell), as can be seen in Supplementary Fig. 1. A 4 K high-resolution Keyence VHX-7000 optical microscope with the depth-composition mode was used to capture the images from top view using the objective lens of 700×. The optical cell was charged at 2.5 mA cm^−2^ (lateral current density) at 25.8 °C using a potentiostat (Gamry Instruments). Images were taken at a frequency of 1 min per frame.

### Electrochemical characterization

Graphite/μ-Si composite working electrodes (diameter 15 mm), Li counter electrode, Celgard 2325 separator (diameter 19 mm) were assembled into CR2032 coin cells (crimped at 560 tonnes m^−^^2^ (800 psi) in an Ar-filled glovebox at 26 °C, with H_2_O and O_2_ content less than 0.5 ppm). Sixty microlitres of 1 M lithium hexafluorophosphate (LiPF_6_) in 30 vol.% ethylene carbonate and 70 vol. % ethyl methyl carbonate + 2 wt.% vinylene carbonate (VC) + 15 wt.% fluoroethylene carbonate (Sigma-Aldrich, purity ≥99.5%, prepared within 2 months, stored in aluminium containers in Ar-filled glovebox at 26 °C, with H_2_O and O_2_ content less than 0.5 ppm, transferred by polypropylene pipette tip) were used as the liquid electrolyte solution. The assembly of the Li||CBD coin cells followed the same procedure. Cyclic voltammetry was performed at a scan rate of 0.1 mV s^−1^ in a potential range of 0.005–1.0 V versus Li|Li^+^. Potentiostatic EIS was conducted within a frequency range of 10 mHz to 1 MHz with an a.c. signal of 10 mV. Ten points were taken per decade. After being charged to the targeting SOC at each step, the cells were relaxed at open circuit potential for 3 h before the EIS measurement was conducted. The lithiation test of the CBD electrode was conducted using a CC–CP protocol with a lithiating current density of 0.028 mA cm^−2^ and a potential cut-off of 5 mV. All electrochemical experiments were carried out under ambient laboratory conditions, with the environment air-conditioned to 25.8 °C. The raw data acquired from the EIS measurements are used for the DRT analysis using the open-source MATLAB code DRTtools available in the literature^34^. The inductance and diffusion frequency regimes are not included in the fitting of the raw data. The regularization parameter was set to 0.01 for all fittings. All electrochemical tests were performed on at least three cells to ensure consistency and reproducibility, with single-cell data representing the average of multiple cells plotted in this study. The specific capacity reported in this study is based on the total mass of the composite working electrode, including the current collector and non-electrochemical active materials.

### Electrolyte solution uptake of the CBD electrodes

The 100% CBD electrodes were cut into 2 × 10 mm^2^ strips and then immersed in the electrolyte solution for 6 h. After being rinsed by DMC for 30 s and dried in a glovebox overnight, the treated CBD electrode was examined by scanning electron microscopy and compared with the untreated CBD electrode. No inert-atmosphere transport sample holder was used.

### X-ray nano-CT, 4D synchrotron X-ray CT and data processing

X-ray nano-CT of the pristine and cycled CBD was performed using a Zeiss Xradia Ultra 810. The cycled CBD electrode after first lithiation was extracted from the coin cell, which was opened in the glovebox using a coin cell decrimper (Hohsen). The electrode was rinsed by DMC for 30 s to remove residual LiPF₆/solvent, and then taken out from the glovebox after overnight drying. The cycled CBD was brittle and easily peeled off from the copper current collector, where a tiny piece of approximately 100 μm was glued onto the tip of a stainless-steel pin under the optical microscope and mounted onto the sample stage for X-ray nano-CT scanning. No inert-atmosphere sample holder was used during transport, as air exposure did not affect the scanning results. A total of 1,400 projections were acquired over a 180° rotation, with an exposure time of 60 s per frame, using a quasi-parallel beam at 5.4 keV. The resulting voxel resolution was 64 nm. CBD was not exposed to X-ray radiation during the lithiation process. A 1/32” PEEK cell, with 1.5 mm wall thickness of the housing was used for the synchrotron X-ray CT, conducted at Beamline I13-2, Diamond Light Source, UK. The electrodes (8.5 wt.%, 17 wt.% Si, 35 wt.% Si and 5 wt.% SiO_*x*_) were cut to 0.85 mm diameter and stacked with Li foil; a 250-μm-thick glass fibre separator was placed vertically so that the X-ray beam transmitted in-plane for each layer. The representativeness of the cycling performance using the operando PEEK cell is compared with coin cell in Supplementary Fig. 22 and discussed in Supplementary Note 14. A pco.edge 5.5 camera with an objective lens of 10× was used to achieve an effective pixel size of 0.325 μm and a field of view of 0.83 × 0.7 mm^2^. A total of 2,000 projections were collected for each tomographic scan, at a temporal resolution of 1.5 min. The data were collected and reconstructed on-site with the built-in algorithm developed at the beamline. Electrochemical cycling between each scan was conducted in the 0.3–0.4 mA cm^−2^ current density range remotely outside the experiment hutch using a Gamry potentiostat (Gamry 1010). Microstructural characterization of the reconstructed 3D data was carried out in commercial software Avizo V9.5 (Avizo, Thermo Fisher Scientific,). The marker-based watershed algorithm^47^ was used to segment the 3D microstructure into different phases. The segmentation uncertainty of Si particles and CBD, by comparing with the ground-truth manual segmentation using a representative volume, is 16% and 13%, respectively, with an offset of ±1 pixel locating at the phase boundaries. Tortuosity factor *τ*_*c*_ was measured by conducting diffusion simulation within the segmented porous structure in the electrode as the flow domain using Avizo Xlab Diffusion^48^ (ThermoFisher Scientific). A gradient of electrolyte concentration was applied between the inlet and outlet to drive the mass flow *Q*_*p*_. The effective mass transport parameter $\frac{\varepsilon }{{\tau }_{c}}$ was obtained by dividing *Q*_*p*_ by *Q*_*e*_ (flow in the volume with porosity *ε* = 1). Mathematics and schematics are introduced in Supplementary Note 15 and Supplementary Fig. 23. For the tortuosity characterization of CBD, only two X-ray scans (that is, pristine and final state) were conducted for the CBD cell, so a linear decreasing trend of porosity *ε*_CBD_ and tortuosity *τ*_CBD_ were assumed based on the homogeneous microstructure of the CBD. These values are embedded in the computational domain (the cloudy phase in Fig. 3c,d) to obtain *τ*_electrode_.

### Digital image and volume correlation

Digital image and volume correlation were carried out using the 2D optical microscopic data and 3D X-ray CT data, respectively. The commercial software Davis v11 (LaVision) was used to extract the displacement field and strain map within the electrode as a function of SOC. The data were divided into patches of regular size (depending on the feature size of interest), and the displacement and strain were measured by tracking the movement of the morphological features represented by groups of intensity values within each patch. Details of the operating principles and the algorithms are detailed elsewhere^43^. A multistage image registration process was used, where each stage uses progressively smaller correlation window sizes, from coarse pass (128 voxels, detecting bulk deformation) to precise pass (48 voxels, detecting local deformation). This method improves displacement convergence and spatial resolution while maintaining robustness against noise. The final correlation window is 48 voxels, with 50% overlap (step size) to increase the spatial resolution, and the average correlation score is approximately 0.91 for DVC and 0.95 for DIC. The deconvolution of the electrode strain into Si and graphite strain from the DIC data was achieved by the following procedure: the optical RGB images of the composite electrode were first segmented into Si, pore and graphite phases using machine learning-based segmentation algorithm by open source software Ilastik. Two binary images (0 for background and 1 for the object of interest), one containing labelled graphite particles and the other labelled Si particles, were each pixel-wise multiplied (that is, masked) with the strain map from the DIC results to extract the strain associated with each phase.

## Online content

Any methods, additional references, Nature Portfolio reporting summaries, source data, extended data, supplementary information, acknowledgements, peer review information; details of author contributions and competing interests; and statements of data and code availability are available at 10.1038/s41565-025-02027-7.

## Supplementary information

Supplementary Information Supplementary Figs. 1–23 and Notes 1–15.

Supplementary Video 1 Video showing the electrode expansion during the 1^st^ full lithiation.

Supplementary Video 2 Video showing the strain during the 1^st^ full lithiation.

Supplementary Video 3 Video showing the electrode expansion during the 2^nd^ full lithiation.

Supplementary Video 4 Video showing the strain during the 2^nd^ full lithiation.

Supplementary Video 5 Single Si particle encapsulated by surrounding graphite particles.

Supplementary Video 6 Volume expansion of 17 wt.% Si composite electrode during 1^st^ lithiation.

Supplementary Video 7 Volume expansion of graphite/SiO_x_ composite electrode during 1^st^ lithiation.

Supplementary Video 8 Electrode expansion associated with CBD evolution.

## Source data

Source Data Figs. 3–6 Statistical source data for the plots in Figs. 3–6.

## Data Availability

Source data are provided with this paper. The other data presented in this study are available from the corresponding authors upon reasonable request.
