# Supplementary material for: Unravelling electro-chemo-mechanical processes in graphite/silicon composites for designing nanoporous and microstructured battery electrodes
Source: Nat Nanotechnol. 2025 Oct 24;20(11):1656–66. doi: 10.1038/s41565-025-02027-7 (PMC12623239; doi:10.1038/s41565-025-02027-7)
Supplement: Supplementary file 1 — Supplementary Figs. 1–23 and Notes 1–15. [file 41565_2025_2027_MOESM1_ESM.pdf]

nature nanotechnology

Supplementary information

https://doi.org/10.1038/s41565-025-02027-7

Unravelling electro-chemo-mechanical processes in graphite/silicon composites for designing nanoporous and microstructured battery electrodes

In the format provided by the authors and unedited

Table of Contents

Supplementary Figures 1-23

Supplementary Notes 1-15

References

Supplementary Figure 1-23

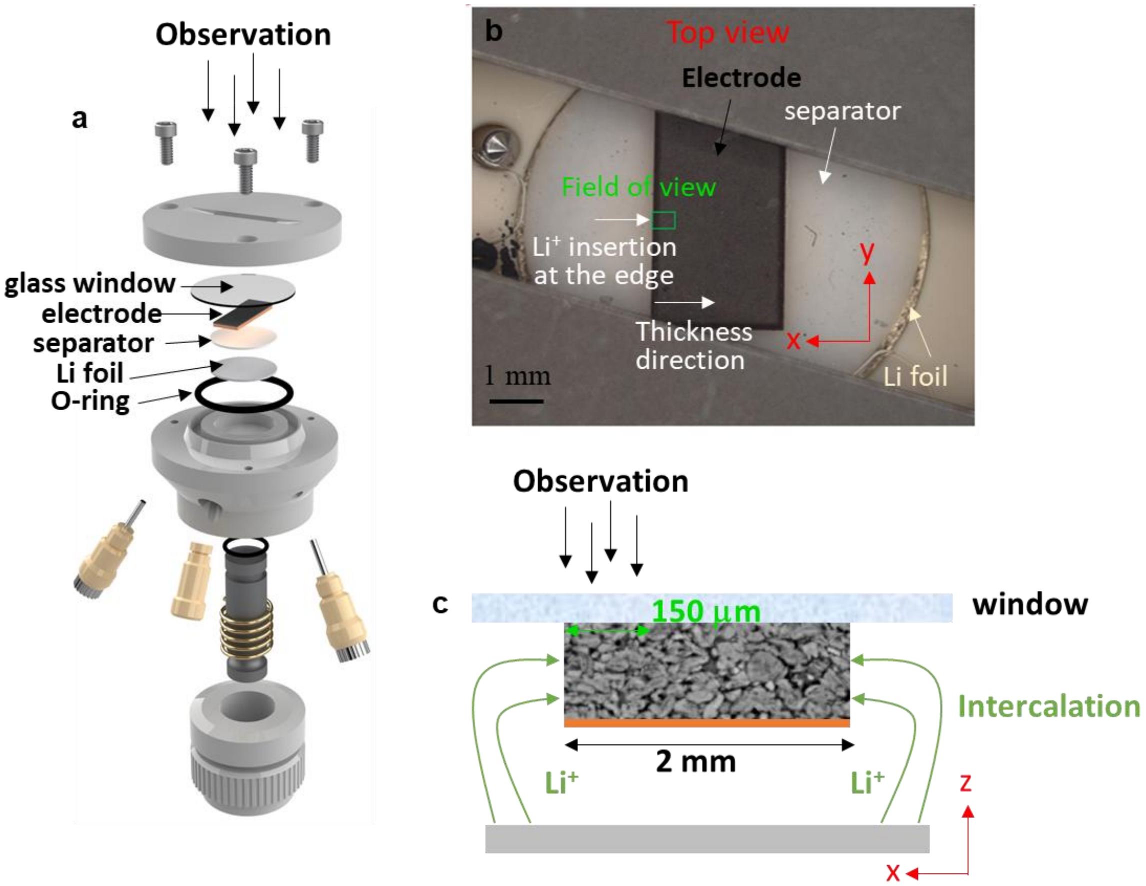

Supplementary Fig. 1: Experiment setup of the operando microscopy measurements. (a) Schematic representation of the assembly of the electrochemical optical cell; (b) Photographic picture of the top view of the geometry of the electrode and the lithiation direction; (c) Schematic representation of the lateral view of the test setup. This experiment setup shown in (a) and (b) is identical to the experiment in our previous publication[2].

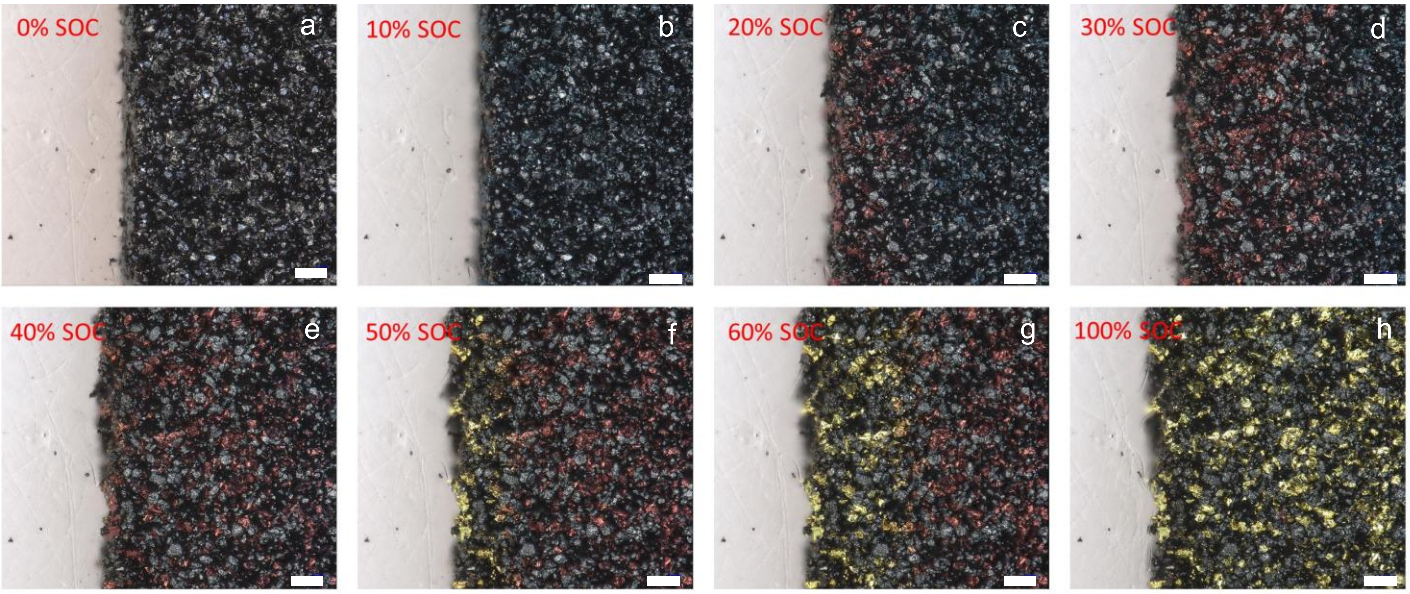

Supplementary Fig. 2: (a) – (g) Multiple optical microscopy images showing the incremental microstructural evolution of the graphite/Si composite electrode from 0% to 60% SOC and at (h) 100% SOC. The optical colour of the graphite phase changes as a function of SOC (grey: 0%; red: 50%; gold: 100%) during the charging process at a lateral current density of 2.5 mA cm^{-2}. Scalebar: 30 μm.

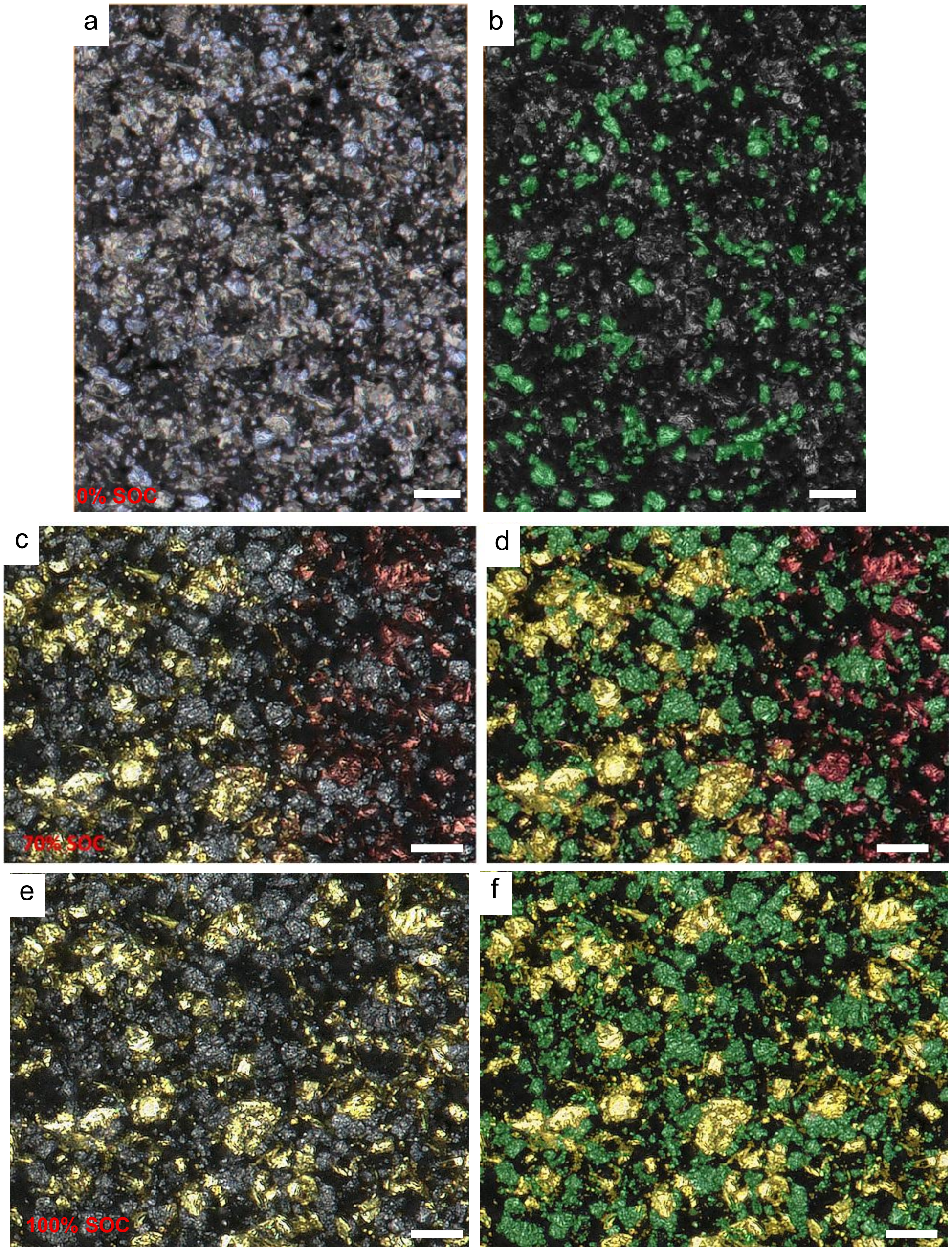

Supplementary Fig. 3: Visual comparisons of the raw optical microscopy images (a), (c), (e) and the corresponding segmentation results (b), (d), (f). In the segmented data, green: Si; red: graphite at Stage II; gold: graphite at Stage I. Scalebar: 20 μm.

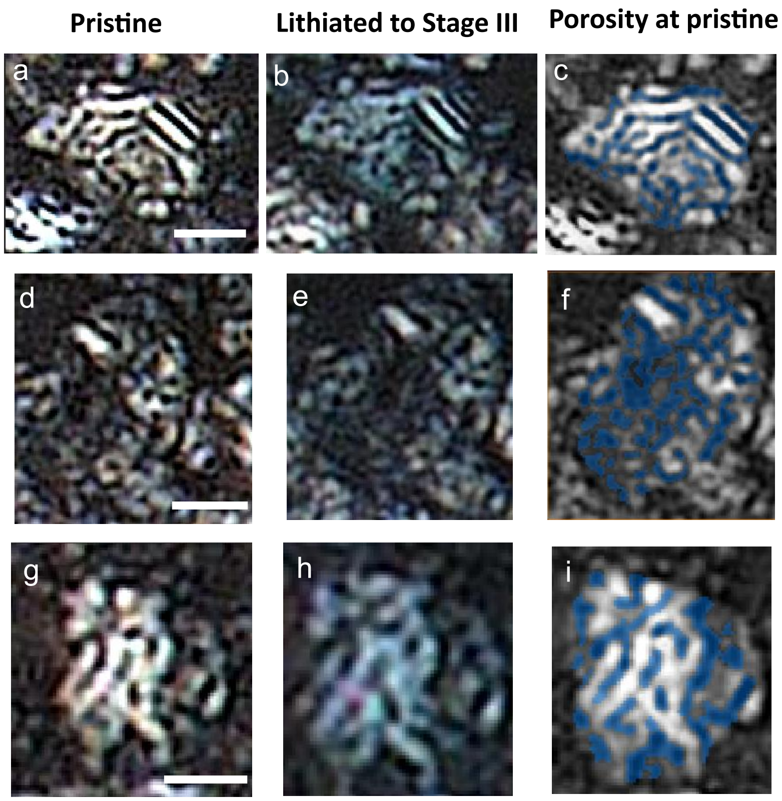

Supplementary Fig. 4: Three exemplary graphite particles that show internal porosities. (a), (d), (g) raw optical images of the graphite particles at 0% SOC; (b), (e), (h) corresponding colour change at Stage III; (c), (f), (i) segmented porosity at 0% SOC. Scalebar: 5 μm.

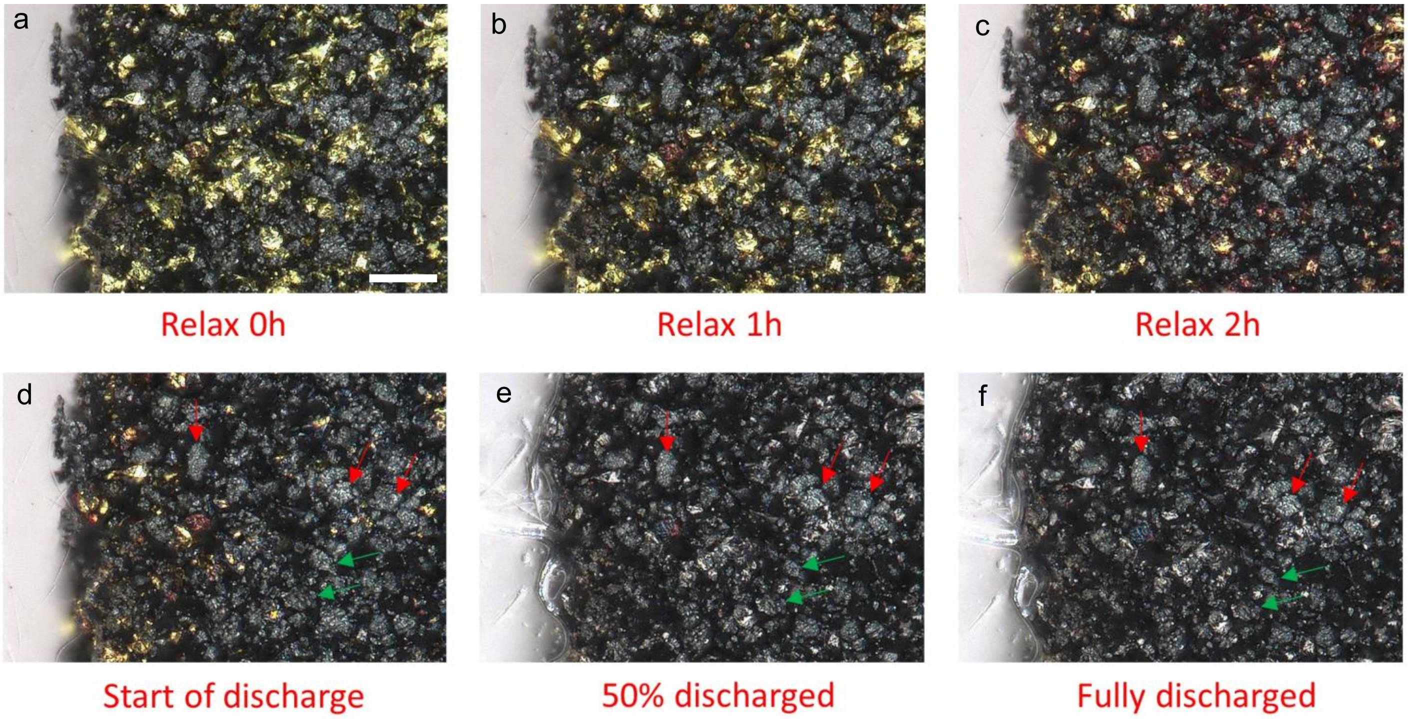

Supplementary Fig. 5: Optical microscopy images showing the relaxation process (a) at the start, (b) after 1 hour and (c) after 2 hours following the 1st (formation) charging of the electrode; (bottom) the 1st discharge process. Exemplary inactive and active Si particles are indicated by the red and green arrows, respectively. Scalebar: 30 μm.

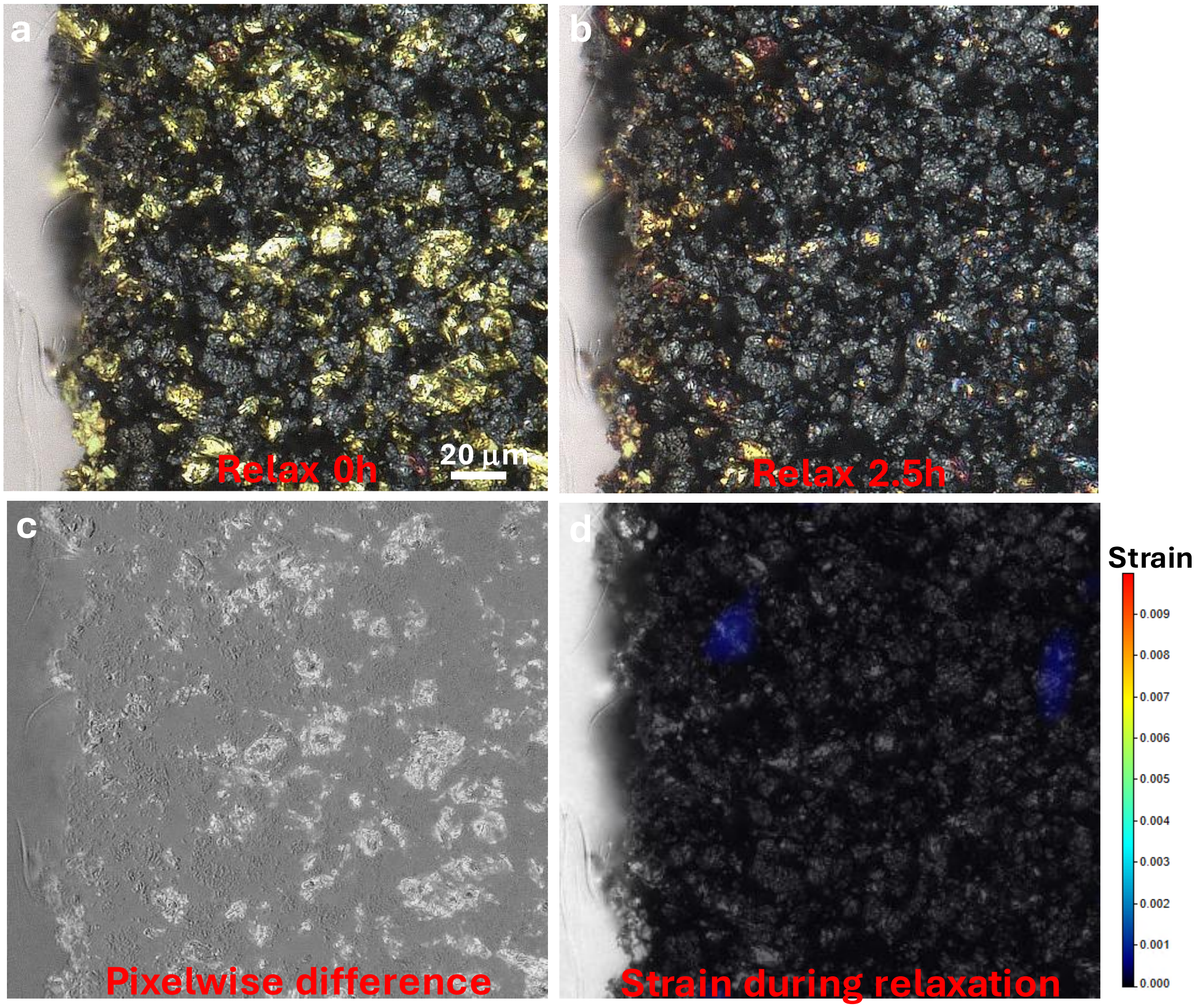

Supplementary Fig. 6: Optical microscopy images showing the morphological change during relaxation at OCP. (a) Timestep at the start of relaxation; (b) after relaxation for 2.5 hours; (c) a pixelwise difference between (a) and (b), where only the colour change of graphite particles is noted; (d) little strain is developed in the Si particles during relaxation.

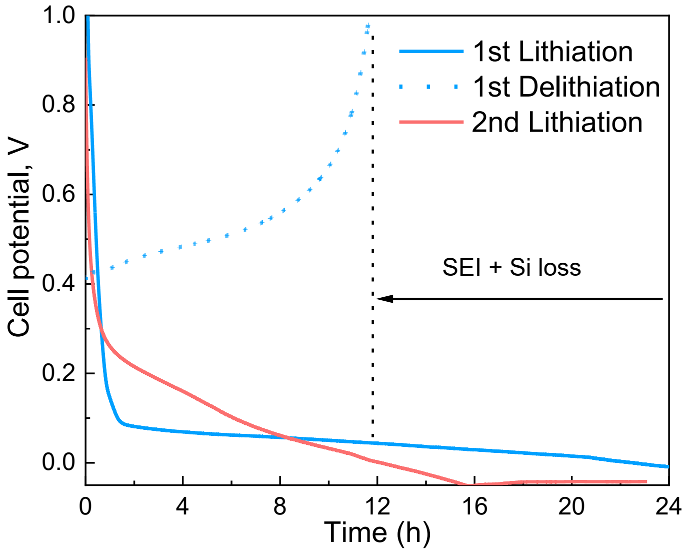

Supplementary Fig. 7: The cell's potential profiles for the 1st and 2nd lithiation processes of the graphite/μ-Si composite electrode in the operando optical experiment.

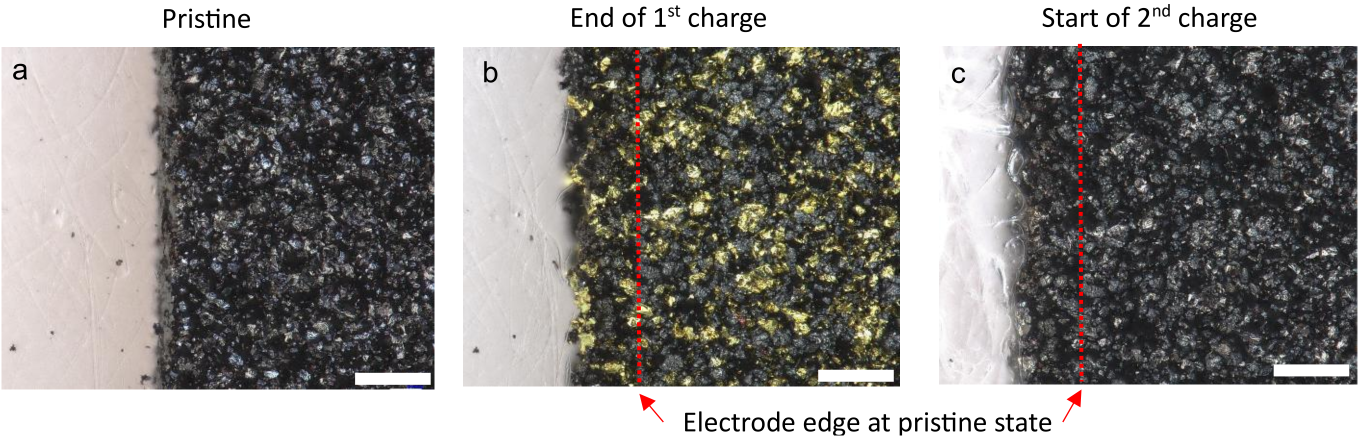

Supplementary Fig. 8: Optical microscopy images showing the particle morphology and electrode expansion (a) before lithiation, (b) at the end of first (formation) lithiation and (c) the start of second lithiation. Porosity recovery is observed from (b) to (c). Scalebar: 50 μm.

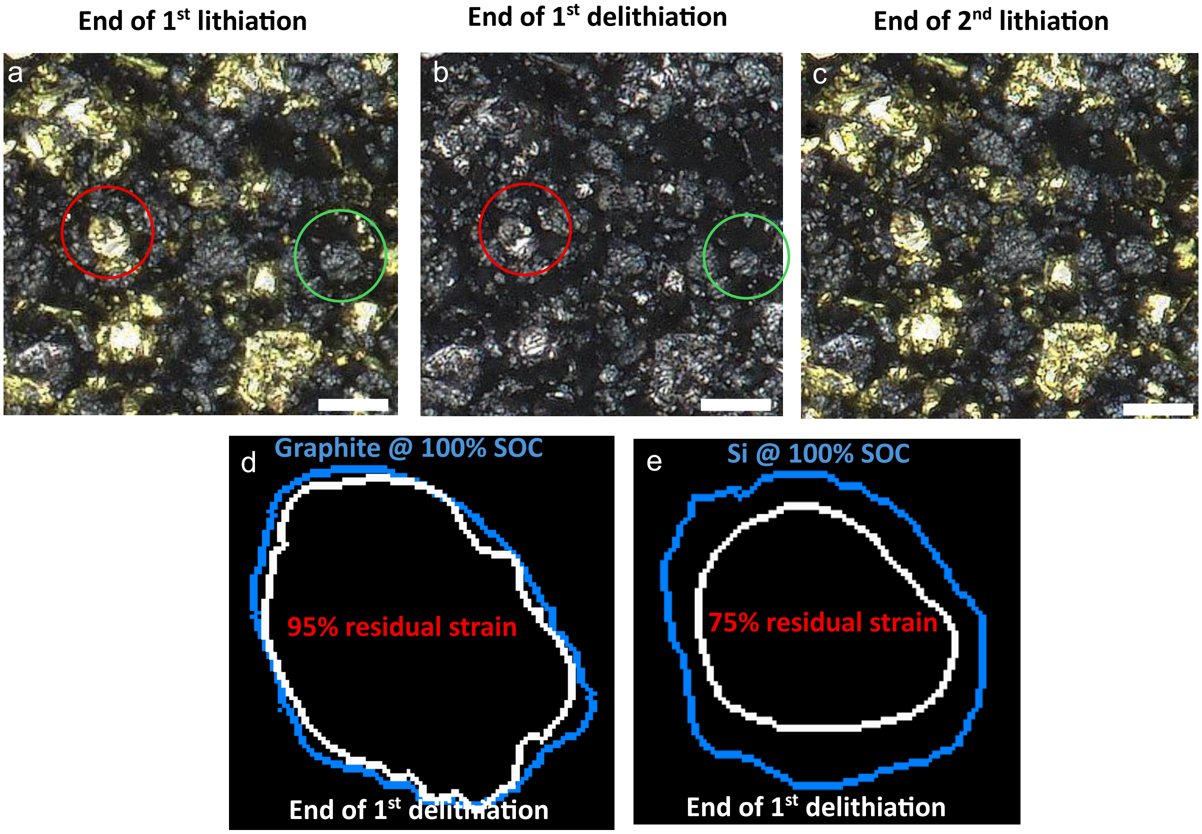

Supplementary Fig. 9: Raw optical images showing the electrode morphologies (a) at the end of 1st lithiation (b) at the end of 1st delithiation and (c) at the end of 2nd lithiation. Exemplary particles that remain active during the process are highlighted in red (graphite) and green circle (Si). (d) and (e) visualize the residual strain of the active graphite (red circle) and Si particles (green circle), and the restoration of porosity after 1st delithiation.

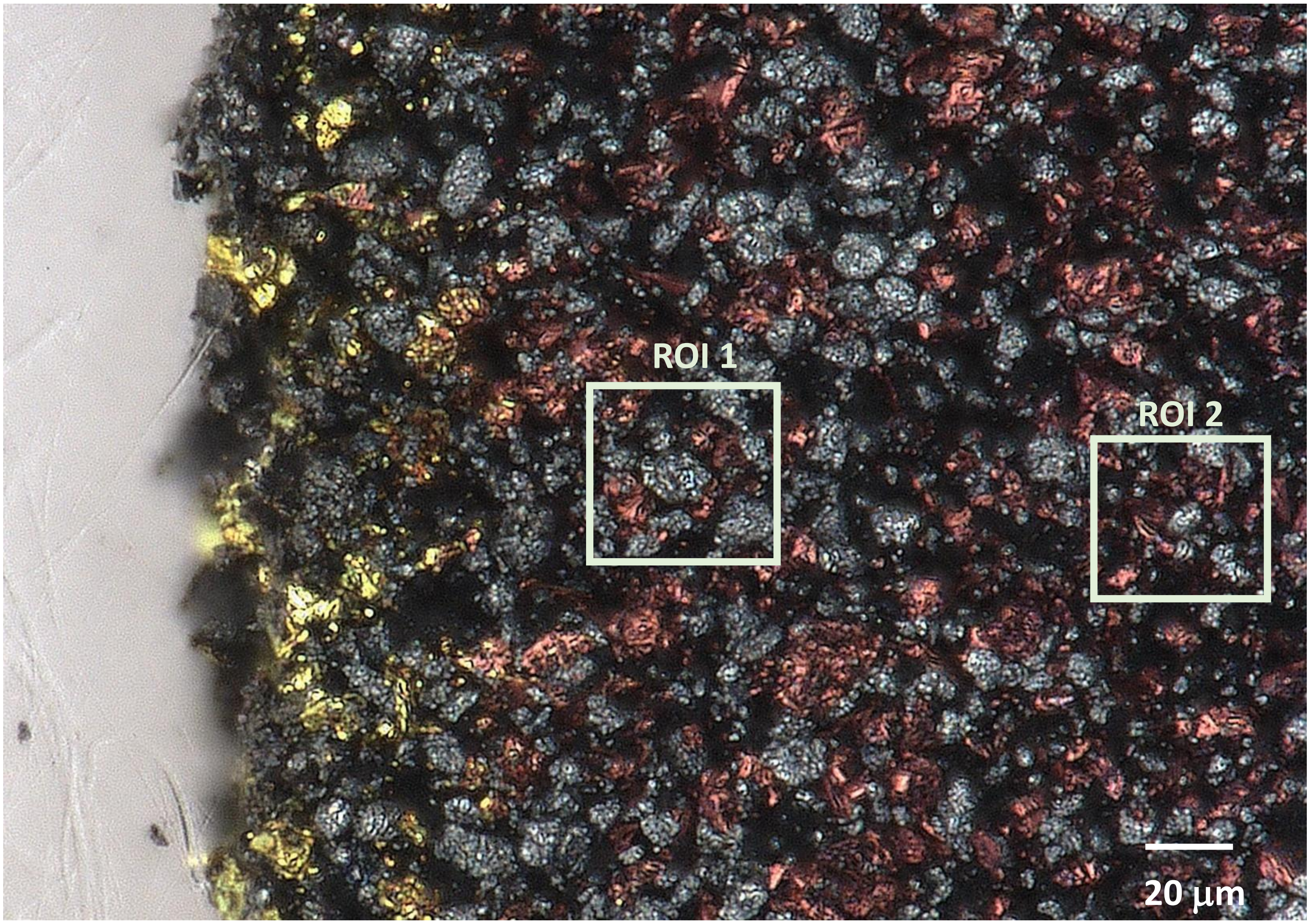

Supplementary Fig. 10: Extracting 2 ROIs for the particle-level analysis as indicated in Fig. 2 in the main text.

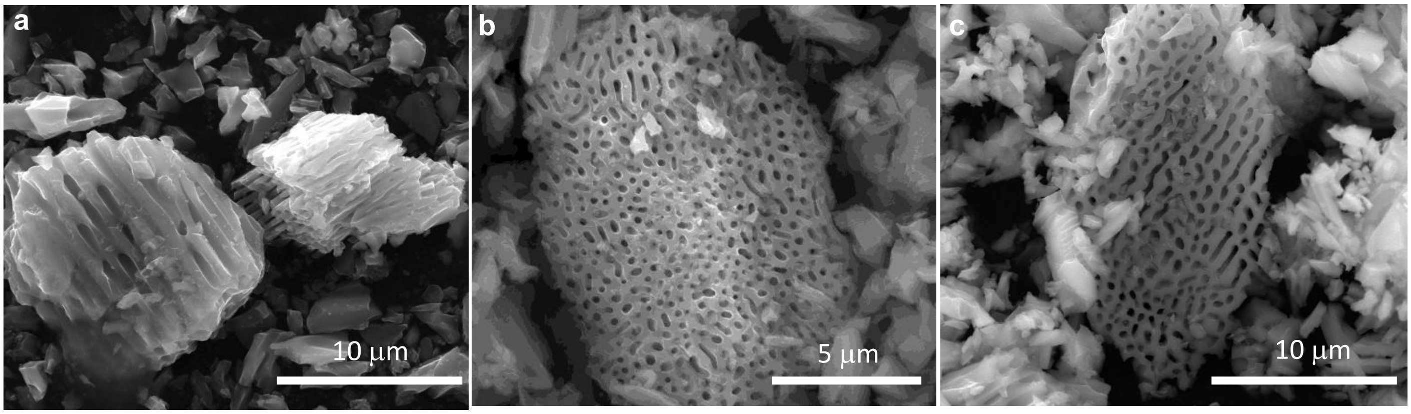

Supplementary Fig. 11: SEM images showing the directional porosity of the Si particles. Panels (b) and (c) indicate two types of porous morphologies viewed from the xy-plane in Fig.2a.

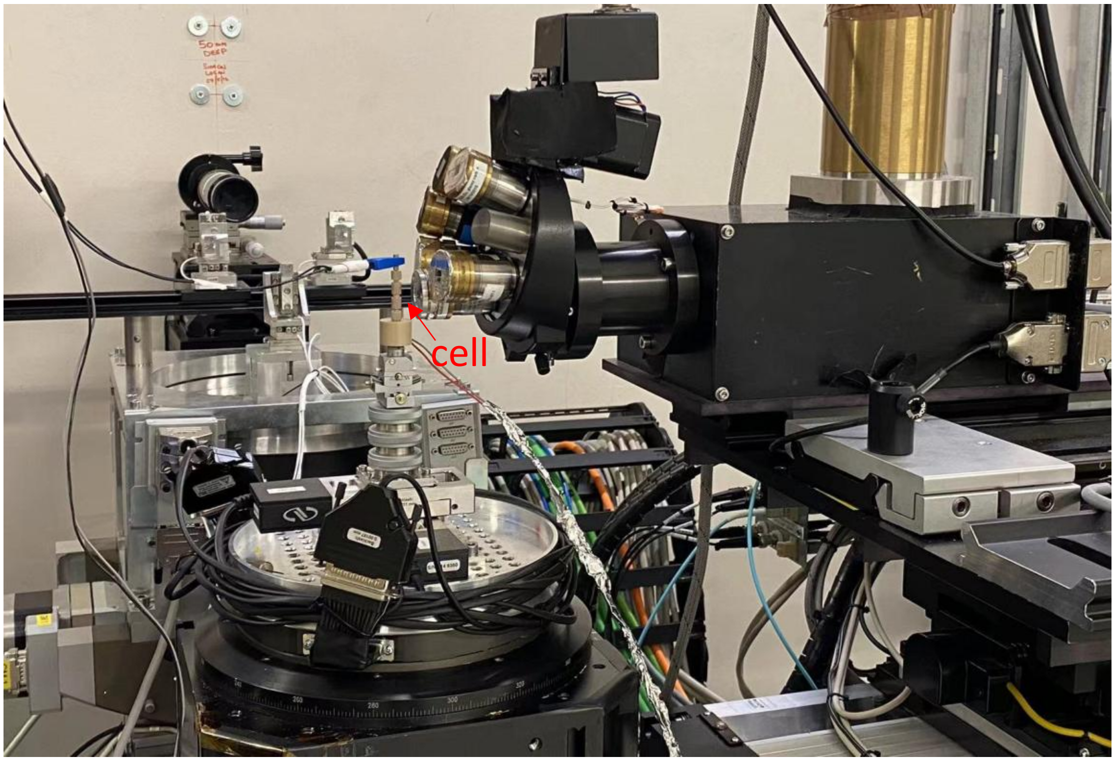

Supplementary Fig. 12: Photographic picture of the synchrotron 4D X-ray CT experimental setup.

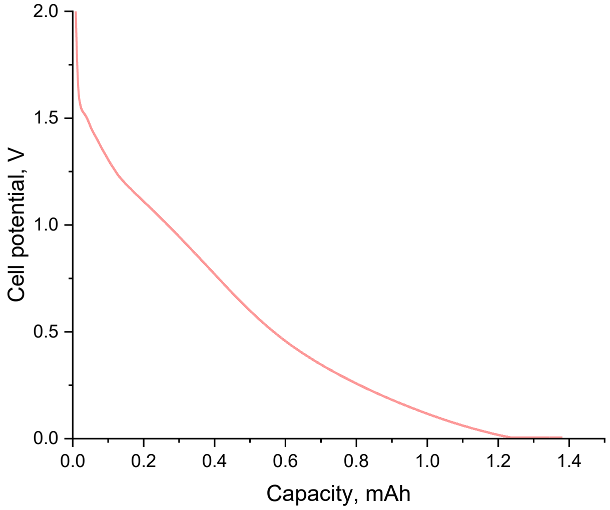

Supplementary Fig. 13: Potential profile of a Li||CBD coin cell during the first lithiation process. The areal and volumetric capacity is measured to be 0.7 mAh cm^{-2} and 350 mAh cm^{-3} respectively, including lithiation and SEI formation, corresponding to 14% total capacity in the composite electrode with 20 wt.% CBD.

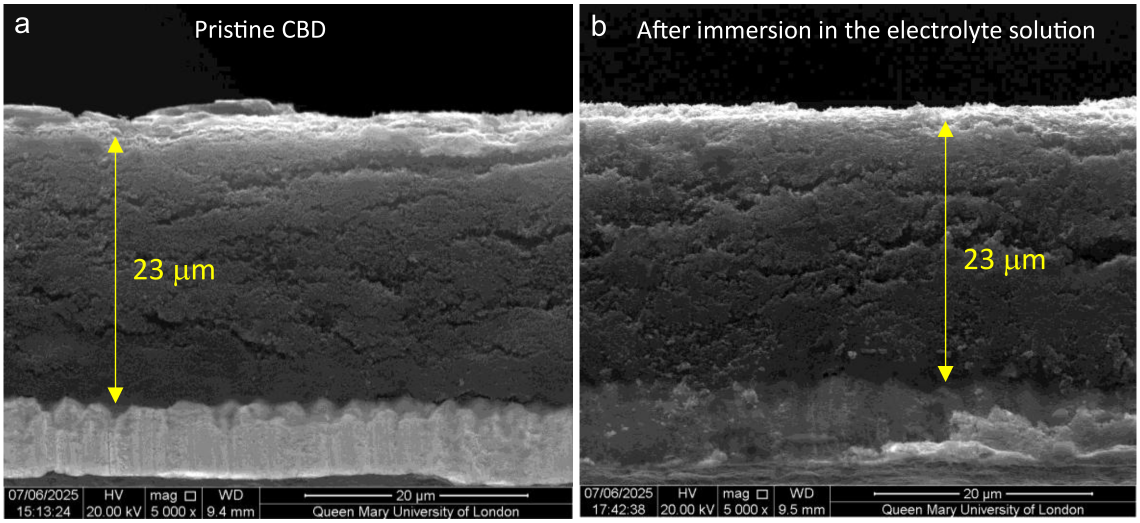

Supplementary Fig. 14: Comparison of CBD thickness under (a) pristine state and (b) after immersion in the electrolyte solution (duration: 6 hour, at 26 ℃ in Ar-filled glovebox) and being rinsed with DMC (duration: 30 second for one time, at 26 ℃ in Ar-filled glovebox).

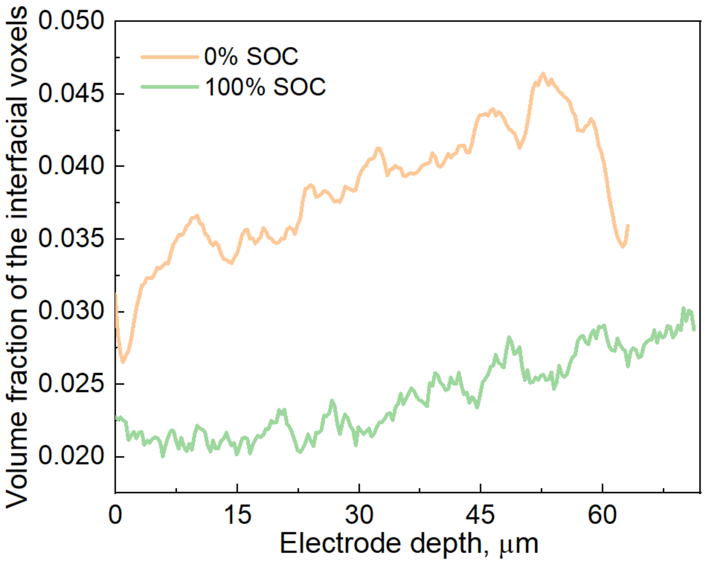

Supplementary Fig. 15: Quantification of the interfacial contacts between graphite particles in the graphite/μ-SiO_x composite electrode.

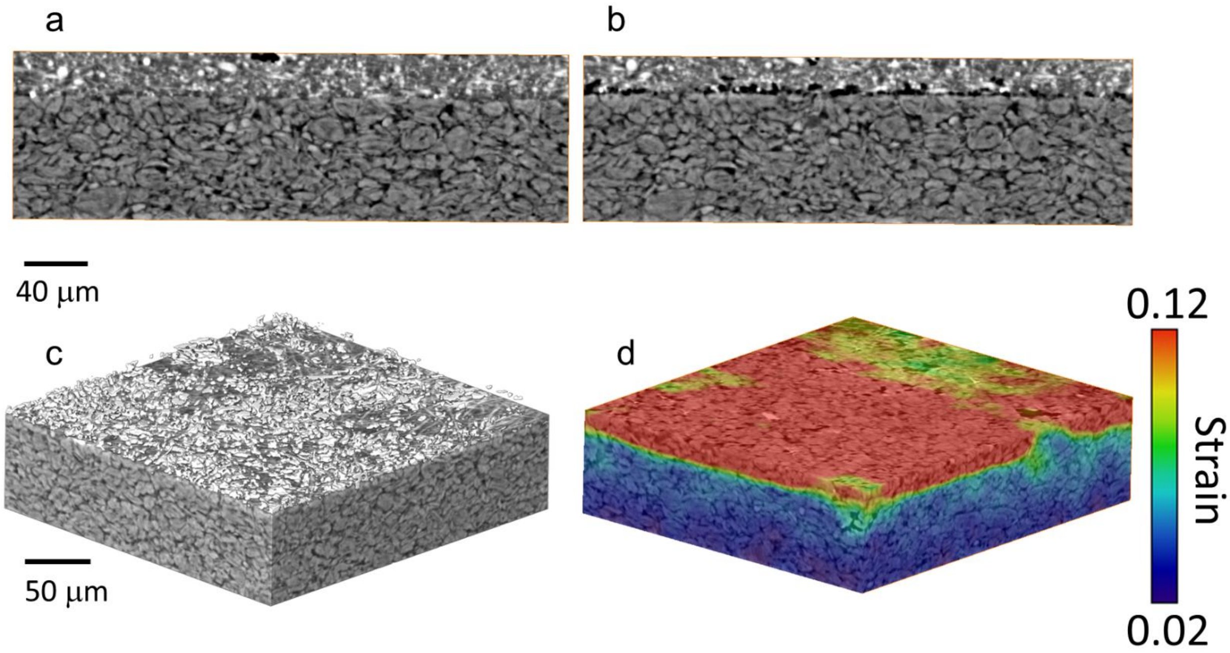

Supplementary Fig. 16: In-plane cross-sectional slice (x-y) showing the lithiation heterogeneity due to size.

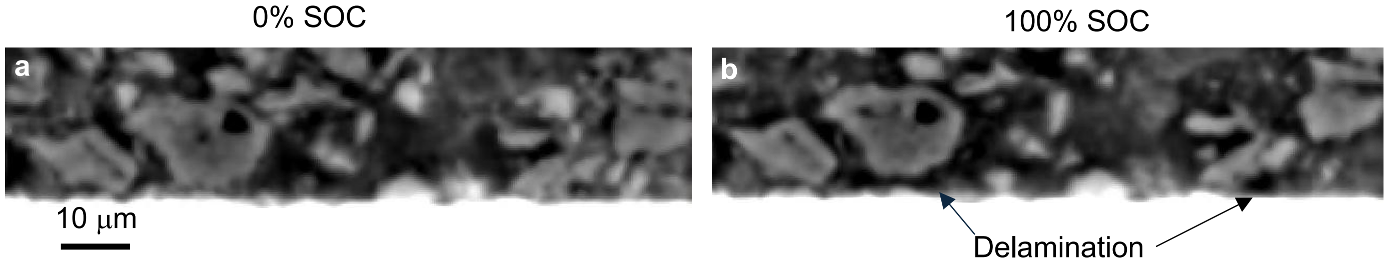

Supplementary Fig. 17: Evidence of delamination at the current collector.(a) A cross section of the electrode before lithiation; (b) the same slice showing the fully lithiated electrode, where particles exhibit delamination from the current collector (saturated white region at the bottom), as pointed out by black arrows.

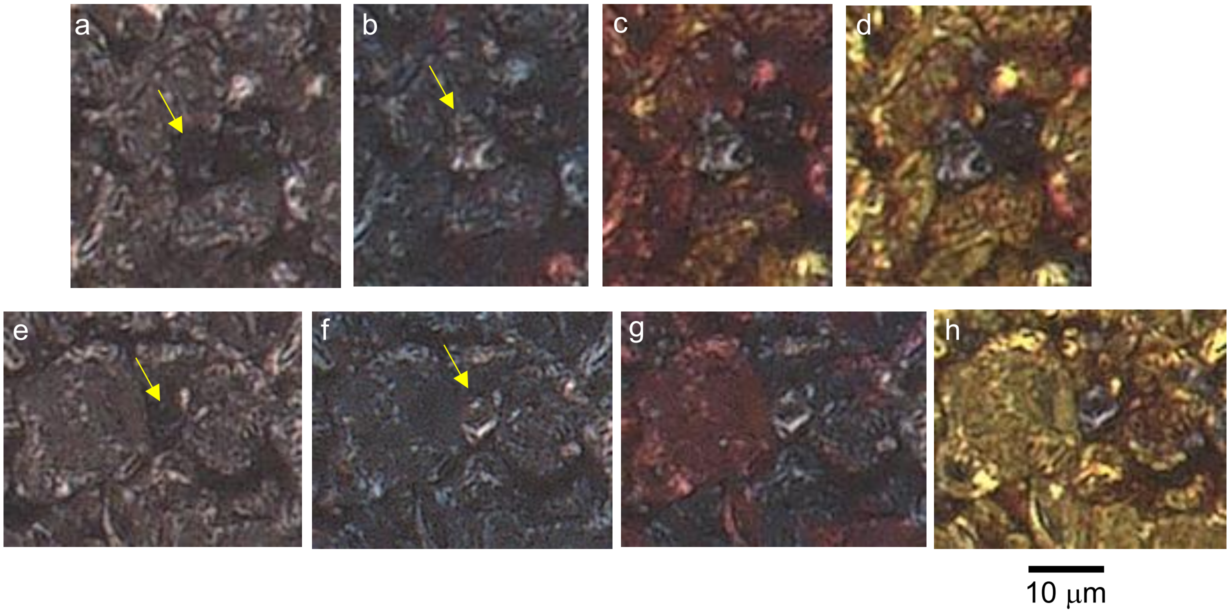

Supplementary Fig. 18 Lithiation kinetics and particle expansion in graphite/SiO_x composite electrode. (a)-(d) and (e)-(h) exhibit two exemplary SiO_x particles (pointed out by yellow arrows) that complete expansion at (b) and (f) respectively, before graphite particles reaching Stage II.

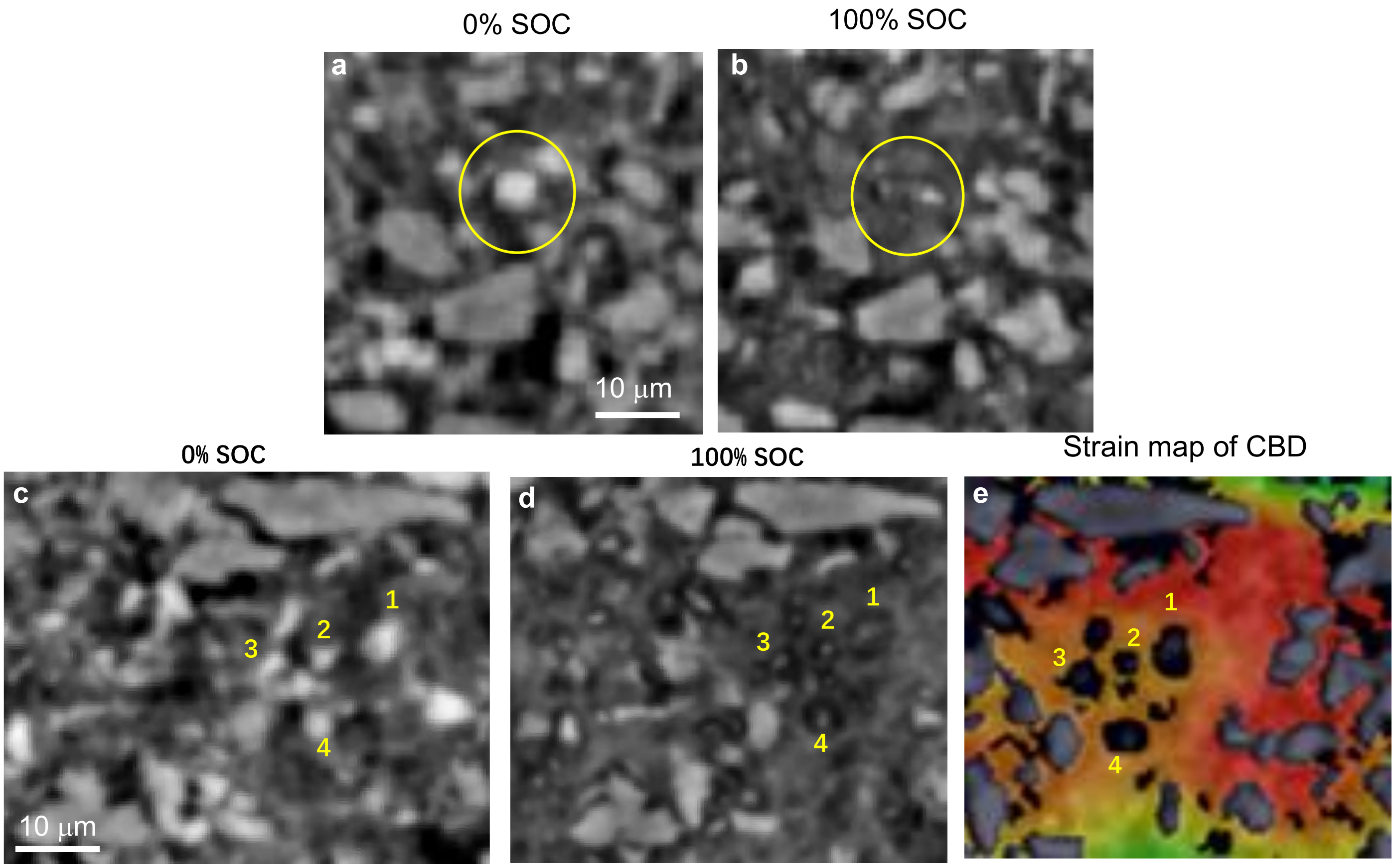

Supplementary Fig. 19: Comparison of the contrast change of the Li_xSi particles. (a) and (b) shows gray Li_xSi phase whilst the four representative Si particles in (c) exhibit dark grey/black Li_xSi phase in (d), appearing as the shell of the central crystalline Si particles. The strain map in (e) corresponds to the segmented CBD domain that does not include the Li_xSi in Particle 1 to 4.

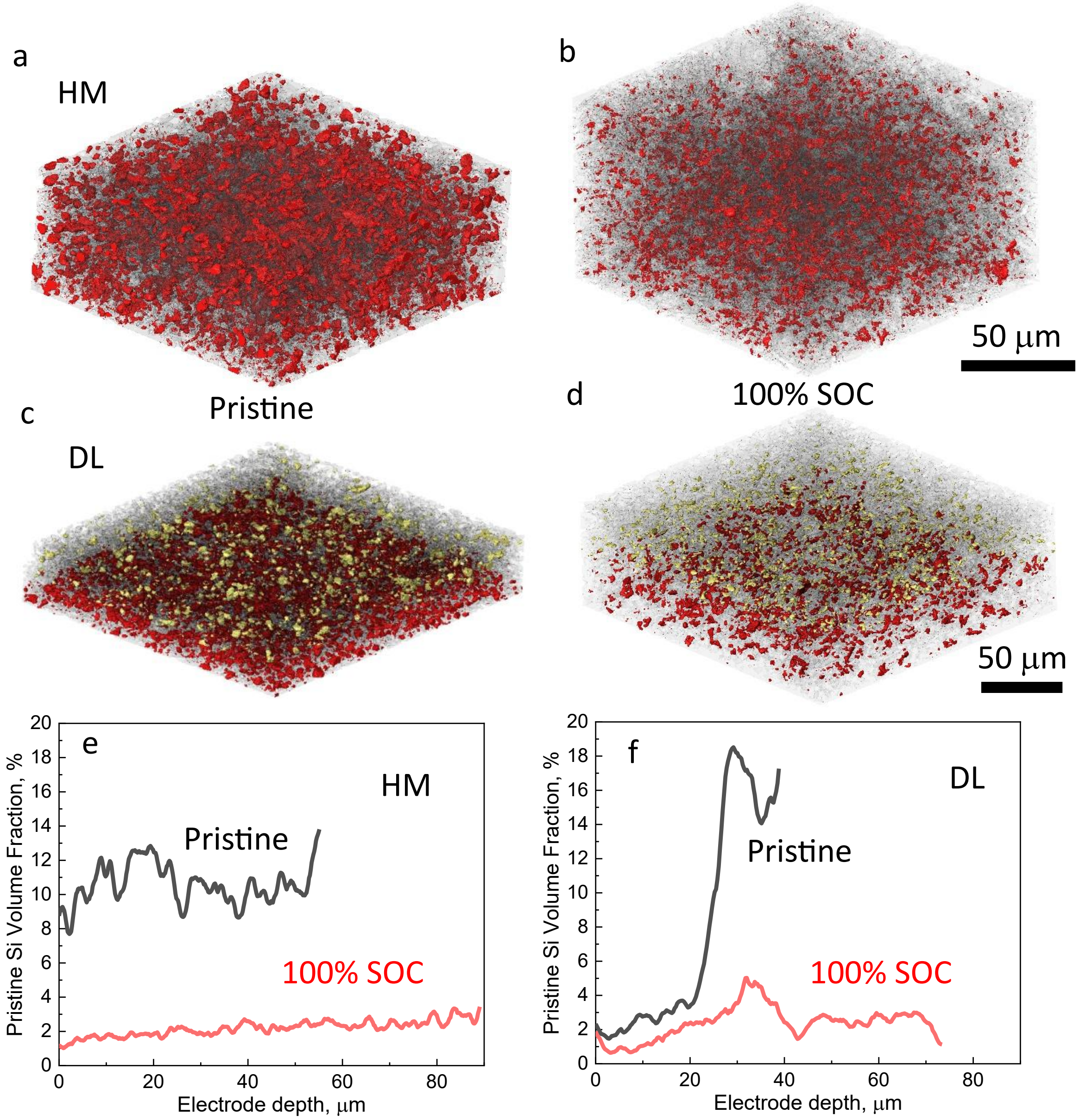

Supplementary Fig. 20: Comparison of the pristine Si before and after full lithiation in the (a), (b) homogeneous (HM) electrode and (c), (d) double-layer (DL), respectively. The volume fraction of the crystalline Si (pristine) particles across the thickness of the HM and DL electrode before and after the lithiation is shown in (e) and (f) respectively.

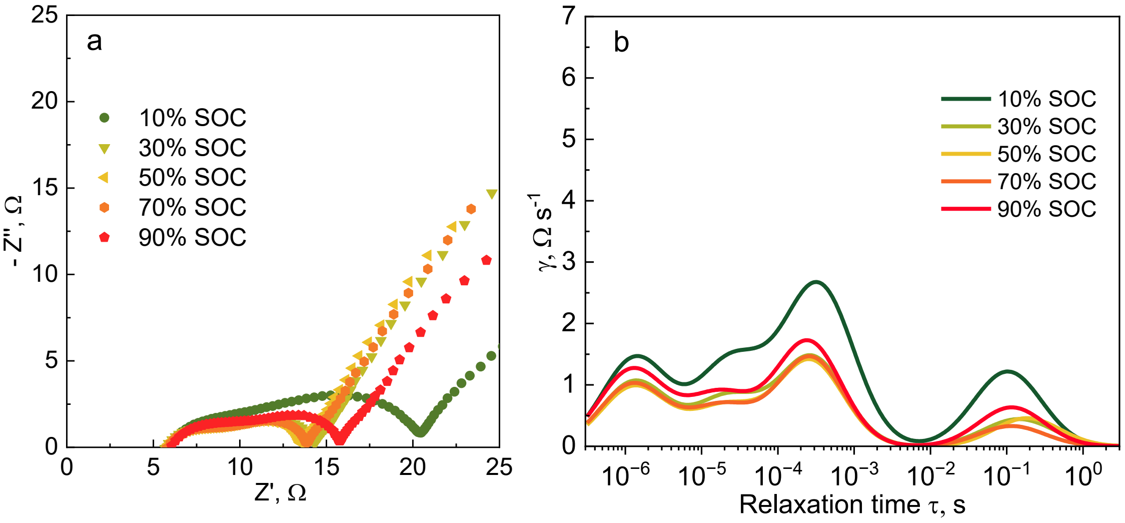

Supplementary Fig. 21: Nyquist plot (a) and DRT peak profiles (b) of the Li||DL coin cell in the 23rd cycle. The cell was cycled using CC-CP protocol at 0.9 mA cm^{-2} within a potential window of 5 mV to 1.5 V. Test was run at 25.8 °C.

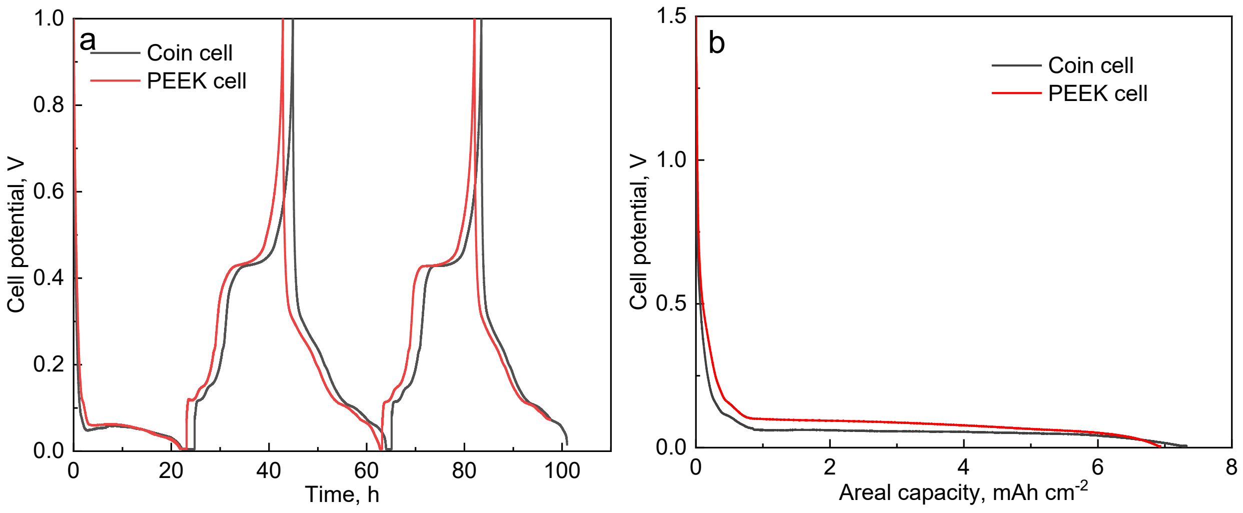

Supplementary Fig. 22 Comparison of the cycling performance between the Li metal coin cell and the operando PEEK cell for the (a) graphite/μ-Si (17 wt.% Si) working electrode and the first lithiation plateau in the cell using the (b) DL working electrode.

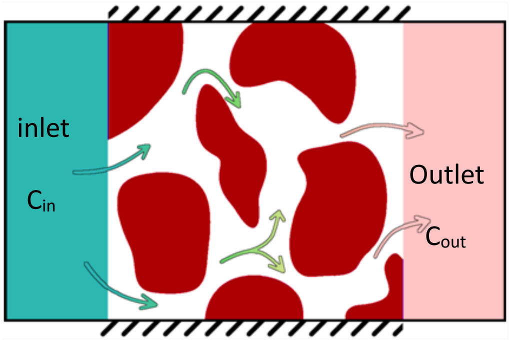

Supplementary Fig. 23: Schematic of the diffusion simulation to obtain the tortuosity factor, where the inlet means the entrance of the simulation domain where high concentration of electrolyte is; the outlet is the exit of the flow domain. The flow is driven by the concertation difference of the fluid at the inlet and outlet, corresponding to C_{in} and C_{out}.Green and pink areas schematically represent reservoirs that contains fluid with distinct concentration; white area and dark red area schematically represents porous region and solid particles respectively.

Supplementary Notes 1-15

Supplementary Note 1: Experiment setup of operando optical microscopy measurements and SOC estimation

The size of the electrode is 2 × 10 mm^2, from where a field-of-view (FOV, 200 × 250 μm^2) is selected for high-resolution inspection (see the experimental setup in Supplementary Fig. 1). Such large size difference causes a full lithiation of the FOV at 0.25% global SOC, which is not informative for the interpretation of electrochemistry. Thus, a local SOC within the FOV is used in the context of this section. Since the lithiation starts from the lateral surface of the electrode (i.e., left-hand side of the FOV), and the full lithiation of graphite always precedes Si particles (judged by the colour changes of graphite that represents staging[1,2] (dark blue (Stage III), red (Stage II), and gold (Stage I)) vs. the expansion of Si particles), the 100% SOC (local) is estimated based on the completion of volume expansion in the Si particles at the right end of the FOV. In this study, the lithiation and delithiation process of the composite graphite/Si working electrode is referred to as 'charging' and 'discharging', respectively.

Supplementary Note 2: Segmentation of optical data

In Supplementary Fig. 2, the raw optical images (left column) are shown with their corresponding segmentation results (right). At the pristine state, an HDR image of the electrode was taken, which helped to better separate the Si and graphite particles due to the enhanced colour difference (Si particles naturally exhibit a blue tint, see Supplementary Fig. 3a). The segmented Si particles are labeled green, and the graphite particles are labelled red (Stage II) and gold (Stage III) to be consistent with their optical colours after lithiation. By comparing raw and segmented images, the accuracy of the segmentation is fairly good. Particularly, at 0% SOC, the majority of the Si particles in the raw images have been picked up in the segmented image, showing a volume fraction of 16%. The segmentation was conducted using a machine-learning-based method in the Ilastik software, which reports that the average probability of the segmentation is approximately 0.85. Note that the CBD was not segmented from the pore phase in optical images, because they are not distinguishable from each other due to the similar black colour with poor contrast. Since the optical experiment only focuses on the microstructural evolution and strain of the solid phases, it is not an issue.

For a 45 wt.% : 45 wt.% ratio between Si and graphite, the theoretical volume fraction of Si should be higher than what is measured (i.e., 16%), using the measured volume fraction of graphite as a reference (36%). However, the graphite particles used in this study are highly porous (see Supplementary Fig. 4). The second column of Supplementary Fig. 4 demonstrates the color change of the same particles during lithiation, confirming their identity as graphite rather than Si. In the third column of Supplementary Fig. 4, the measured porosity of these graphite particles averages 39%. This represents open porosity, as it is measured from the particle surface. It is well established that synthetic graphite exhibits a closed-to-total porosity ratio exceeding 20%[3,4]. Thus, the total porosity of the graphite particles may be estimated as $0.39/(1-0.20) \approx 0.487$. It should be noted that meso- and nano-pores below the imaging resolution (0.15 μm) are not captured in this measurement. With this conservative porosity estimate, the expected volume fraction of Si is calculated to be 0.237, still higher than the measured 16%.

This discrepancy may be attributed to the following factors: (1) Some Si particles may be obscured from the surface view or buried within the CBD; (2) A single 2D image may not provide fully accurate quantification of the overall volume fractions. Despite this, nearly all visibly identifiable Si particles are included in the segmentation data. Even if a few Si particles were missed, it means the Si particle strain presented in Extended Data Fig. 1b is based on 70% (16% divided by 23%) of the total Si particles, which are visible and large enough to be representative, and the strain analysis based on these segmented Si regions remains valid.

Supplementary Note 3: Lithium exchange during relaxation at the open circuit potential

Following the 1st lithiation, the electrode is held at open circuit potential (OCP) for relaxation. During this stage, the intercalated lithium in graphite starts to equilibrate, as indicated by the fading of Stage I (gold) (Supplementary Fig. 5). No morphological changes are observed in the Si particles, as is evidenced by the negligible pixel-wise difference (Supplementary Fig. 6c) and minimal strain (Supplementary Fig. 6d) between the beginning and end of relaxation, except for the colour changes of graphite particles (appear as white in Supplementary Fig. 6c). This suggests little lithium exchange between the graphite and Si particles during relaxation.

Supplementary Note 4: Factors that affect coulombic efficiency in the operando optical Li metal cell

The inactive 'dead' Si particles during the 1st delithiation process are due to their isolation from the electrically conducting network (Supplementary Fig. 5). As shown in Supplementary Fig. 7, the initial cell's coulombic efficiency (ICE) is just about 50% in cells having a high Si loading (45 wt.%) composite electrode, caused by the SEI formation, degradation and loss of active Si particles due to the isolation from electrical contact and structural damage after expansion/contraction. Besides, a large mechanical irreversibility (i.e., residual strain) at the electrode level is observed (Supplementary Fig. 8), mainly related to the specific setup of the optical experiment, where the electrode can expand freely in the lateral direction, as no compressive constraint is implemented, as in coin and pouch cells, which can lead to reduced electrical contact, more severe particle cracking that generates fresh surfaces, and consequently, more extensive SEI growth[5,6].

At the particle scale, the high initial irreversible capacity can be further understood by examining the measured residual strain in both graphite and Si phases, as shown in Extended Data Fig. 1b and Supplementary Fig. 9. Active Si particles exhibit a residual strain of 75% after the first lithiation/delithiation cycle (formation cycle), indicating that a significant portion of lithium involved in the alloying reaction becomes irreversibly trapped within the core of the structurally damaged Si particles. This structural degradation—and the associated irreversible capacity loss—can be mitigated by limiting the maximum SOC during cycling. As shown in Fig. 2e, crack initiation can occur as early as 65% SOC. Further lithiation beyond this point can lead to cumulative particle damage, resulting in lower coulombic efficiency and shortening the cell's cycle life.

In contrast, graphite particles show even higher residual strain (~95%), yet their electrochemical reversibility remains intact. This is evidenced by the reversible colour change of graphite particles observed optically—from gold (SOC = 100%) to grey (SOC = 0%) and back to gold in the second cycle. The high residual strain in graphite is likely due to its intra-particle porosity, which accommodates its volume changes and dissipates the elastic energy/stress. No apparent structural damage is observed in graphite particles, as their expansion is significantly less detrimental than that of Si particles.

Supplementary Note 5: Residual strain of graphite and Si particles

After the first delithiation, graphite particles exhibit higher residual strain (95%) than Si particles (85%, or 75% if "dead" Si particles from the second cycle are excluded; see Supplementary Fig. 9), which is pointed out by red and green circle respectively in Supplementary Fig. 9. Both are electrochemically active with little sign of decohesion. The low strain reversibility in graphite is attributed to its stable planar structure, internal porosity (> 40%) and relatively small volume change. In contrast, the strain irreversibility in Si particles arises from structural damage. This also explains why Si-based electrodes are often cycled with a capped SOC (approx. 70%) to preserve structural integrity.

Supplementary Note 6: Possible unresolved sub-micron Si particles in the CBD

Although micron-sized Si particles are used in this study, it is acknowledged that some unresolved sub-micron Si particles may be present. However, given that the Si used has a D10 of approximately 3 µm, the majority of particles are visible and successfully segmented (accounting for 5% of the volume in the 17% Si electrode). Any unresolved sub-micron Si particles, if present, would contribute a volume fraction well below 0.5% (i.e., 10% of the 5% total), which is negligible compared to the CBD volume fraction of 35–40%. It is therefore reasonable to conclude that this uncertainty does not affect the interpretation of CBD expansion, which is supported by X-ray nano-CT imaging of the pure CBD sample (Fig. 3g to 3j).

Supplementary Note 7: Quantitative analysis of the evolution of microstructural parameters

Fig. 3o and p compare the volume fraction of the pristine Si particles upon lithiation along the depth direction in two electrodes with different Si mass loadings (8.5% vs. 17%). The result shows that a fraction of Si particles does not undergo lithiation; the lower the Si content, the more thorough the Si utilization is. Fig. 3q depicts the variation of macro-porosity, CBD porosity, total electrode porosity (including CBD porosity) and CBD volume fraction as a function of SOC. The macro-porosity decreases linearly from 0.31 (0% SOC) to 0.22 (100% SOC), and the volume fraction of CBD increases from 0.39 (0% SOC) to 0.51 (100% SOC), yielding a drop of the total electrode porosity from 0.47 (0% SOC) to 0.32 (100% SOC). Consequently, this leads to an increase in the electrode tortuosity factor $\tau_{electrode}$ from 2.3 (0% SOC) to 5.15 (100% SOC) as presented in Fig. 3r. Besides, the effective mass transport parameter, $D_{eff} = \frac{\varepsilon}{\tau}$, is compared between the macro-pore phase, CBD and the electrode (after accounting for the CBD porosity). $D_{eff\_electrode}$ is 15% - 20% larger than D_{eff_CBD}, underscoring the impact of the macro-porosity. Notably, $D_{eff\_macro\text{-}pore}$ is 20% - 25% lower than $D_{eff\_electrode}$, highlighting the influence of the nano porosity in CBD, which is often neglected in microstructural characterization and continuum modelling. This quantitative analysis parameterizes the effective mass transport property of the electrode to deliver Li^+ ions as the microstructure evolves, and is crucial for the continuum modelling of graphite/μ-Si composite electrode towards the improved reliability and confidence, particularly as highlighted by the distinct difference between $D_{eff\_electrode}$ and $D_{eff\_macro\text{-}pore}$.

Supplementary Note 8: Electron transport-induced underutilization of SiO_x particles

While high porosity supports ion transport, it compromises electronic connectivity, especially near the separator, leading to underutilized SiO_x. To support this, Fig. 4e(i)-(iii) visualize the spatial distribution of the pristine SiO_x particles at different SOCs. Substantial SiO_x underutilization is seen at the end of charge, which is quantitatively analyzed in Fig. 4e (iv). Initially, three SiO_x peaks appear at 15, 30, and 56 µm. By 100% SOC, Peak 3 (closest to the current collector) lithiates most, followed by Peak 2, while Peak 1 remains largely inactive—confirming electron transport as the limiting factor.

Supplementary Note 9: In-plane electrode strain vs. depth profiles for different electrodes

Extended Data Fig. 2 reveals that in the graphite/μ-SiO_x electrode, strain rises rapidly before 40% SOC and then slows down due to transport limitations and preferential lithiation near the separator. In the graphite/μ-Si electrode, strain increases steadily with SOC and shows minimal gradient, with a sharp drop in deeper regions from current collector delamination. The distinct trends of strain vs. SOC illustrated in Extended Data Fig. 2 are reflected macroscopically in Fig. 5d.

Supplementary Note 10: Volumetric strain distribution in graphite and CBD across thickness

The heterogeneous strain between individual graphite particles as a function of the particle size (symbol size) and the position is shown in Extended Data Fig. 3. An incremental in-plane and through-thickness variation as a function of SOC is observed. Note that the average volumetric strain in graphite particles at 100% SOC is consistent with the result measured by the DIC (Extended Data Fig. 1b). However, the DIC result shows a higher strain at 60% SOC. This is caused by a higher effective charging current in graphite particles due to the higher Si mass loading between the two experiments (optical: 45 wt.% vs. X-ray CT: 17 wt.%). The CBD shows a similar trend and magnitude of the strain as graphite particles. Both optical measurements and synchrotron X-ray CT consistently show a graphite strain of 0.2 to 0.25 at 100% SOC. Meanwhile, the CBD strains measured by nano-CT in a pure CBD sample and by synchrotron X-ray CT in the composite electrode are consistent. It is speculated that this similarity occurs because both graphite and CBD are predominantly carbonaceous materials, and thus may undergo comparable volumetric changes during lithiation. While graphite expands primarily along the c-axis, each graphite particle is polycrystalline, and therefore the overall particle expansion tends to be isotropic. On the other hand, the conductive carbon (C45) in the CBD has a disordered structure, and its expansion is attributed to a combination of surface adsorption, interlayer swelling, and pore filling. These mechanisms may compensate for the structural differences between layered graphite and amorphous carbon, leading to a similar net strain. Additionally, solvent uptake by the CMC binder also contributes to CBD swelling.

It is also noted that CBD within the orange circular regions in Fig. 5g exhibits slightly higher strain (0.22 to 0.25) than that in the blue circular region (0.2), which may be attributed to the presence of Si particles within this area that drives a higher ionic and electronic fluxes, leading to enhanced surface adsorption, interlayer swelling, and pore filling locally. However, it is unlikely that the presence of Si particles introduces errors in the local strain measurements within the CBD. As shown in Fig. 5k, the lithiated Si particles (i.e., Li_xSi) within the orange circles do not induce any apparent strain aberration in their vicinity. Furthermore, the expanded Li_xSi phase (dark grey or black) is not misidentified as CBD during segmentation, indicating that the local CBD strain is unaffected by possible phase mislabelling. The presence of Si particles does not affect the strain of graphite particles. From a thermodynamic perspective, lithiation of Si and graphite occurs concurrently during the first cycle due to their overlapping lithiation potentials, as evidenced by Figure 1a–c and Supplementary Video 1.

Supplementary Note 11: Over-segmentation of CBD due to the identical grayscale intensity of Li_xSi

The lithiation of Si particles incurs a change of grayscale intensity, from white to gray (Supplementary Fig. 19a and b), or dark gray/black (Particle 1, 2, 3 and 4 in Supplementary Fig. 19c and d). This variation may be associated with different degrees of crystalline-to-amorphous transition in the Li_xSi phase—where darker contrast likely corresponds to a higher lithium content (i.e., a larger value of *x*). The former causes an over-segmentation of Li_xSi into CBD due to the identical grayscale intensity, which can be quantitatively estimated: the initial CBD volume fraction is 39%, which should increase to approximately 39% × 1.25 = 48.75% at 100% SOC, estimated based on the expansion ratio measured from the pure CBD sample via X-ray nano-CT (~25%). However, CT segmentation yields a CBD volume fraction of 52% at 100% SOC, indicating a discrepancy of 52% - 48.75% = 3.25%, which is attributed to mislabelled Li_xSi within the CBD. This corresponds to 3.25% / 52% ≈ 6.25% of the total segmented CBD at 100% SOC. However, some Li_xSi do not fully transform into regions with CBD-like intensity and thus are not mislabelled as CBD, as indicated by the strain map of the segmented CBD (Supplementary Fig. 19e). These particles retain a distinct black shell—indicative of Li_xSi—surrounding a crystalline Si core. This shell creates a sharper gradient in grayscale intensity at the CBD/Li_xSi interface, which serves as a boundary to prevent inundation in the watershed segmentation algorithm, and thereby effectively preserves the phase distinction.

Supplementary Note 12: Peak identification in the dQ/dV plots

Fig. 6k and l show cathodic peaks for amorphous Si (a-Si) to amorphous lithium-Si alloy Li_xSi (a-Li_xSi) (A), and graphite phase transitions: Stage III (B), Stage II (C), and Stage I (D), with corresponding anodic peaks. Besides, the single-phase alloying reaction from a-Li_xSi to a-$Li_{x+y}Si$ (same amorphous lithium-Si alloy as Li_xSi but with higher lithium content) occurs throughout the potential range of 50 mV to 0.2 V[7], contributing to a broad peak. Moreover, a minor cathodic peak (E) at approx. 40 mV in Fig. 6l (less distinct in Fig. 6k) corresponds to the phase transition from a-$Li_{x+y}Si$ to *c*-$Li_{15}Si_4$ (*c* stands for crystalline)[8]. This leads to a delithiation plateau at 0.43 V in Fig. 6i and j and a large peak E' in Fig. 6k and l, representing the phase transition from *c*-$Li_{15}Si_4$ to a-Si.

Supplementary Note 13: Distribution relaxation time (DRT) analysis of the homogeneous (HM) and double-layer (DL) electrode

The Nyquist plot in the Extended Data Fig. 5a shows the raw EIS data of the Li||DL coin cell at 90% SOC at cycle #3 and #23, respectively, which highlights a distinct change in the shape and magnitude of the semi-circles after 20 cycles. To identify different electrochemical processes in the convoluted impedance signals in the complex impedance plot, we employ the distribution of relaxation times (DRT) approach[9,10] to deconvolute distinct polarization sources. This is achieved by extracting a continuous distribution of time constants through spectral fitting with an infinite series of RC elements (Extended Data Fig. 5b). Three major processes are identified in the graphite/μ-Si composite electrode (Extended Data Fig. 5c). Previous study assigned P_1 at high frequency as the electrical contact between the components in the electrode, including active material particles, binder, conductive carbon and current collectors; P_2 and P_3 represent the Li-ion diffusion through the SEI layers and the charge transfer reaction respectively[11,12]. Extended Data Fig. 5d-f displays the evolution of the DRT profiles at incremental cycling numbers in the cell containing the HM electrode. It is observed that the polarization remains stable within the first 8 cycles and then increases drastically at the 13th cycle. The sharp increase of P_1 implies the deterioration of the electronic conducting network, leading to uneven supply of electrons. This could cause over-lithiation of active materials at high SOC, as reflected by the substantial increase of P_3. In comparison, the cell with the DL electrode shows a similar polarization at the beginning and then becomes much lower as the cycle number increases (Extended Data Fig. 5g-i). It is noted that the cell with the DL electrode displays a decreasing trend of polarization, which might arise from the fragmentation of the Si particles that are conducive to faster kinetics. These fractured Si particles remain electrochemically active thanks to the double-layer structure for two reasons: (1) a high volumetric concentration of the CBD (10 wt.% within approx. half of the volume); (2) the graphite layer at the top acts as the buffer to restrict the expansion from the Si-dense layer at the bottom. These two factors contribute to the intact electrical percolation during cycling.

The evolution of different polarization resistance is quantified by integrating the area under each peak, as shown in Extended Data Fig. 5j-l. The DL electrode has a high contact resistance at low SOC and then decreases as the charging proceeds. This could be attributed to the higher compressive force (e.g., better electrical contact) as the electrode expands. A drastic increase in contact resistance is seen in the cell with the HM electrode at cycle #13, with an exponential trend towards high SOC, which might be caused by the loss of electrical percolation because of the microstructural degradation. Besides, Extended Data Fig. 5k delineates that the SEI resistance of the cell with the DL electrode stays lower than that of the one with HM, and it initially decreases and then increases after 70% SOC. We assume that the initial reduction of SEI resistance arises from the mechanical degradation and even fracture of the formed SEI as the Si particles expand during charging, followed by a regeneration process that causes the increase at higher SOC[11]. The drastic increase in the cell with the HM electrode in the 13th cycle may be caused by the low local electrolyte concentration due to the reduced porosity.

Notably, the SEI resistance is decreasing with the cycle numbers in the cell with the DL electrode. This is primarily attributed to two factors: (1) the progressive formation of new electrochemically active surfaces resulting from the mechanical fracturing of Si particles during lithiation and delithiation; (2) the tendency of neighboring amorphous Li_xSi particles to coalesce, which not only reduces interfacial contact resistance but also effectively expands the electrochemically active surface area and SEI layers connected in parallel, thereby lowering the overall resistance. This mechanism, however, is less relevant in the cell with the HM electrode, where the distribution of Si particles is more dispersed and less densely packed than in the bottom layer of the DL electrode.

Extended Data Fig. 5l shows a stable charge transfer resistance in both cells within the first 8 cycles, with a slightly higher magnitude in the Li||HM one. After the 13th cycle, a substantial drop of the reaction kinetics occurs in the cell with the HM electrode, which could arise from the deteriorated electron conduction, as the trend in Extended Data Fig. 5l correlates well with Extended Data Fig. 5j; it may also come from the over-lithiation of the remaining active particles that leads to surface crowding effect; insufficient electrolyte concentration due to the clogging of porosity could be another source, consistent with the phenomenon in Extended Data Fig. 5k. The Nyquist plot and DRT analysis of the EIS raw data at the 23rd cycle of the Li||DL coin cell are presented in Supplementary Fig. 21 where a low resistance and minimum increase can be noticed compared to that of the 13th cycle, suggesting a beneficial effect of the double-layer composite electrode design in suppressing the cell's capacity fade. It is worth mentioning that this study mainly focuses on the impact of 3D

microstructural design on electrochemical performance, using 0D conductive additive (C45), CMC/SBR binder and untreated Si particles.

Supplementary Note 14: Representativeness of the cycling performance of the operando PEEK cell

The cycling performance of the exemplary composite electrode (17% Si), including two full cycles plus the initial crystalline to amorphous transition stage between the Li metal coin cell and the PEEK cell, is shown in Supplementary Fig. 22a. The result exhibits consistent cycling profiles. In Supplementary Fig. 22b, the potential voltage profiles of the coin and PEEK Li||DL cells during the first lithiation process are also reported and show good agreement.

Supplementary Note 15: Calculation of the tortuosity factor using continuum CFD simulation

The electrolyte flow driven by a concentration gradient in a fully porous volume is described by Fick's law (Eq. 1) as

$$Q_e = AD\frac{\Delta c}{L} \qquad \text{(Eq. 1)}$$

where D is the diffusivity of electrolyte solution, Δc is the concentration difference, A and L are the cross-sectional area and length of the flow domain. In a porous medium, Eq. (1) is modified as

$$Q_p = \frac{\varepsilon}{\tau_c} AD\frac{\Delta c}{L} \qquad \text{(Eq. 2)}$$

where ε is the porosity and can be estimated by image analysis. Dividing Q_p by Q_e (Eq. 3) gives the effective transport parameter thereby the tortuosity factor,

$$\frac{Q_p}{Q_e} = \frac{\varepsilon}{\tau_c} \qquad \text{(Eq. 3)}$$

References

1 Harris, S. J., Timmons, A., Baker, D. R. & Monroe, C. Direct in situ measurements of Li transport in Li-ion battery negative electrodes. *Chemical Physics Letters* **485**, 265-274, doi:https://doi.org/10.1016/j.cplett.2009.12.033 (2010).

2 Lu, X. *et al.* Multiscale dynamics of charging and plating in graphite electrodes coupling operando microscopy and phase-field modelling. *Nature Communications* **14**, 5127, doi:10.1038/s41467-023-40574-6 (2023).

3 Dresel, E. M. & Roberts, L. E. J. Closed Pores in Synthetic Graphite. *Nature* **171**, 170-170, doi:10.1038/171170a0 (1953).

4 Choi, Y.-J., Lee, Y.-S., Kim, J.-H. & Im, J.-S. Optimization of Pore Characteristics of Graphite-Based Anode for Li-Ion Batteries by Control of the Particle Size Distribution. *Materials* **16** (2023).

5 Šedina, M., Šimek, A., Báňa, J. & Kazda, T. A short review of the effect of external pressure on the batteries. *Monatshefte für Chemie - Chemical Monthly* **155**, 221-226, doi:10.1007/s00706-023-03162-4 (2024).

6 Zhang, K. *et al.* Effects of external pressure on cycling performance of silicon-based lithium-ion battery: modelling and experimental validation. *RSC Advances* **14**, 29979-29991, doi:10.1039/D4RA05354K (2024).

7 Chan, C. K., Ruffo, R., Hong, S. S., Huggins, R. A. & Cui, Y. Structural and electrochemical study of the reaction of lithium with silicon nanowires. *Journal of Power Sources* **189**, 34-39, doi:https://doi.org/10.1016/j.jpowsour.2008.12.047 (2009).

8 Obrovac, M. N. & Christensen, L. Structural Changes in Silicon Anodes during Lithium Insertion/Extraction. *Electrochemical and Solid-State Letters* **7**, A93, doi:10.1149/1.1652421 (2004).

9 Schmidt, J. P., Berg, P., Schönleber, M., Weber, A. & Ivers-Tiffée, E. The distribution of relaxation times as basis for generalized time-domain models for Li-ion batteries. *Journal of Power Sources* **221**, 70-77, doi:https://doi.org/10.1016/j.jpowsour.2012.07.100 (2013).

10 Quattrocchi, E. *et al.* Deconvolution of electrochemical impedance spectroscopy data using the deep-neural-network-enhanced distribution of relaxation times. *Electrochimica Acta* **439**, 141499, doi:https://doi.org/10.1016/j.electacta.2022.141499 (2023).

11 Pan, K., Zou, F., Canova, M., Zhu, Y. & Kim, J.-H. Comprehensive electrochemical impedance spectroscopy study of Si-Based anodes using distribution of relaxation times analysis. *Journal of Power Sources* **479**, 229083, doi:https://doi.org/10.1016/j.jpowsour.2020.229083 (2020).

12 Guo, J., Sun, A., Chen, X., Wang, C. & Manivannan, A. Cyclability study of silicon–carbon composite anodes for lithium-ion batteries using electrochemical impedance spectroscopy. *Electrochimica Acta* **56**, 3981-3987, doi:https://doi.org/10.1016/j.electacta.2011.02.014 (2011).

13 Zhao, Y.-M. *et al.* Advances of polymer binders for silicon-based anodes in high energy density lithium-ion batteries. *InfoMat* **3**, 460-501, doi:https://doi.org/10.1002/inf2.12185 (2021).

14 Yi, R., Zai, J., Dai, F., Gordin, M. L. & Wang, D. Dual conductive network-enabled graphene/Si–C composite anode with high areal capacity for lithium-ion batteries. *Nano Energy* **6**, 211-218, doi:https://doi.org/10.1016/j.nanoen.2014.04.006 (2014).

15 Xu, T. *et al.* In situ synthesis of porous Si dispersed in carbon nanotube intertwined expanded graphite for high-energy lithium-ion batteries. *Nanoscale* **10**, 16638-16644, doi:10.1039/C8NR04587A (2018).

16 Tan, W., Yang, F., Lu, Z. & Xu, Z. A Design Strategy of Carbon Coatings on Silicon Nanoparticles as Anodes of High-Performance Lithium-Ion Batteries. *ACS Applied Energy Materials* **5**, 12143-12150, doi:10.1021/acsaem.2c01655 (2022).
